# Comparison of the mechanical properties of biodegradable and titanium osteosynthesis systems used in oral and maxillofacial surgery

**DOI:** 10.1038/s41598-020-75299-9

**Published:** 2020-10-23

**Authors:** Barzi Gareb, Charlotte C. Roossien, Nico B. van Bakelen, Gijsbertus J. Verkerke, Arjan Vissink, Ruud R. M. Bos, Baucke van Minnen

**Affiliations:** 1 Department of Oral and Maxillofacial Surgery, University Medical Center Groningen, University of Groningen, Hanzeplein 1, 9713 GZ Groningen, P.O. Box 30001, 9700 RB Groningen, The Netherlands; 2 Department of Rehabilitation Medicine, University Medical Center Groningen, University of Groningen, Hanzeplein 1, 9713 GZ Groningen, P.O. Box 30001, 9700 RB Groningen, The Netherlands; 3 Department of Biomechanical Engineering, University of Twente, Drienerlolaan 5, 7522 NB Enschede, The Netherlands

**Keywords:** Bone, Trauma, Preclinical research, Biomedical materials, Implants

## Abstract

To guide the selection of osteosynthesis systems, this study compared the mechanical properties of biodegradable and titanium osteosynthesis systems. SonicPins Rx and xG were subjected to pull-out tests. Additionally, 15 biodegradable (Inion CPS 2.0 and 2.5 mm; LactoSorb 2.0 mm; Macropore 2.0 mm; Polymax 2.0 mm; BioSorb FX 2.0 mm; ResorbX 2.1 mm; Osteotrans-MX 2.0 mm with plate thicknesses 1.0 and 1.4 mm; SonicWeld Rx_plate_/Rx_pins_, xG_plate_/Rx_pins_ and xG_plate_/xG_pins_ 2.1 mm without and with tapping the burr hole) and six titanium (CrossDrive (2006), CrossDrive (2018), MaxDrive; all 1.5 and 2.0 mm) straight, four-hole osteosynthesis systems were evaluated. All systems were subjected to tensile, bending and torsion tests. Pull-out loads of the SonicPins were comparable (P = 0.423). Titanium systems’ tensile loads were higher than biodegradable systems (P < 0.001). CrossDrive (2018) and MaxDrive systems’ tensile and torsional stiffness were lower, accompanied with higher ductility, than corresponding CrossDrive (2006) systems (P < 0.001). Bending stiffness of 1.5 mm titanium systems was comparable to, and of the 2.0 mm systems higher than, all biodegradable systems (P < 0.001). Regarding biodegradable systems, Inion CPS 2.5 mm had highest tensile load and torsional stiffness, SonicWeld 2.1 mm highest tensile stiffness, and BioSorbFX 2.0 mm highest bending stiffness (P < 0.001). On the basis of the results of this study, the CrossDrive (2018) and MaxDrive 1.5 mm titanium systems are recommended for midface fractures (e.g., zygomatic or maxillary fractures) and osteotomies (e.g., Le Fort I osteotomy), and the CrossDrive (2018) and MaxDrive 2.0 mm titanium systems for mandibular fractures and osteotomies when a titanium osteosynthesis system is used. When there is an indication for a biodegradable osteosynthesis system, the SonicWeld 2.1 mm or BioSorbFX 2.0 mm are recommended for midface fractures and osteotomies, and the Inion CPS 2.5 mm biodegradable system for mandibular osteotomies and non-load bearing mandibular fractures, especially when high torsional forces are expected (e.g., mandibular symphysis fractures).

## Introduction

Titanium osteosynthesis systems are currently the systems of choice in oral and maxillofacial surgery. A combination of titanium plates and screws results in excellent mechanical and handling properties, providing adequate bone stability^1^. The disadvantages of titanium osteosyntheses include: palpability^2^, sensitivity to temperature changes^3^, stress shielding of the underlying bone^4^, growth restrictions^5^, interference with radiographic imaging and radiotherapy^4,6,7^, spread of titanium particles in the soft tissue and regional lymph nodes^8^, and possibly mutagenic effects^3^. Consequently, titanium systems are removed in a second operation in 5–38% of cases^9^.

Biodegradable osteosynthesis systems, made of resorbable (co-)polymers, significantly reduce the need to remove implants in a second operation^9^. The other advantages of biodegradable osteosyntheses are: no sensitivity to temperature changes, no interference with radiographic imaging and radiotherapy, no growth disturbances, and a more gradual transfer of stress to the healing bone^5,10–12^. Biodegradable systems have, however, limitations including less favorable mechanical properties compared to titanium systems, a need to tap the screw hole before inserting the screws, and tissue reactions to the prolonged presence of foreign materials^13,14^. These limitations result in higher perioperative screw breakage and longer operation times compared to titanium systems as well as the removal of symptomatic biodegradable systems in up to 17% of the cases^9^.

Recently, new titanium osteosynthesis systems have been introduced to improve perioperative handling (e.g., adjusting the screw head to improve the grip on the screws) and to reduce stress shielding of the underlying bone by adjusting the production process to lower the stiffness of these systems^15,16^. Over 12 different titanium osteosynthesis systems (without taking the different sizes of each system into account) are used currently in oral and maxillofacial surgery (OMF-surgery)^9,17^. The biodegradable systems have also been improved to overcome the limitations of the less favourable mechanical properties, to avoid tissue reactions, and to improve perioperative handling. This was done by adjusting the copolymer composition, by using ultra-sound activated pins whereupon the pinheads fuse with the osteosynthesis plate, and by obviating the need to tap the screw hole. Currently, over 36 different biodegradable osteosynthesis systems are available on the market with different compositions and mechanical properties^13,18^. Yet, due to the presumed less favourable mechanical properties of biodegradable compared to titanium osteosynthesis systems, the use of biodegradable systems is currently restricted to midface or non-load bearing mandibular fracture fixation. Because of the recent improvements in both types of osteosynsthesis systems and the lack of studies comparing these systems, it is still unclear for surgeons which titanium and biodegradable osteosynthesis systems are suitable and prefered for treatment of fractures and fixation of osteotomies.

Examples of improved biodegradable systems are SonicWeld Rx and the recently introduced SonicWeld xG (Gebrüder Martin GmbH & Co., Tuttlingen, Germany)^13^. Both systems use thermoplastic biodegradable pins instead of screws. These pins are inserted into the burr hole using an ultrasound probe, resulting in a flow of the biodegradable polymer into the cancellous bone, which obviates the need to tap the burr hole. This approach has been shown to increase the mechanical properties of the biodegradable osteosynthesis systems^12,19,20^. However, when ultra-sound activated biodegradable pins are only inserted into cortical bone, their axial pull-out strenghts are significantly lower compared to biodegradable screws due to the insufficient retention properties of the smoother cortical bone^12,20^. Therefore, although the burr hole does not normally have to be tapped when applying ultra-sound activated SonicWeld systems, we hypothesized that tapping the burr hole in specific situations (i.e., when only applied in cortical bone) could strengthen the osteosynthesis systems by increasing the contact area and thereby increasing the mechanical retention of the fused pin in the cortical bone layer.

To guide OMF-surgeons and to make recommendations in the selection of osteosynthesis systems, this study aimed to determine and compare mechanical properties of commonly used biodegradable and titanium osteosynthesis systems in OMF-surgery.

## Material and methods

The most commonly used titanium and biodegradable osteosynthesis systems in OMF-surgery were selected^9,17^. The specifications of all the included osteosynthesis systems (i.e., 15 biodegradable and 6 titanium systems), including sizes and compositions, are summarized in Table 1. All the osteosynthesis systems had undergone the sterilization process of the manufacturer and were tested before the expiration date. The mechanical tests were performed six times per system and per application method which corresponds to the American Society for Testing Materials standards (ASTM D638;^21^).

**Table 1:** Specifications of all the included osteosynthesis systems.

Brand name	Manufacturer	Plate composition	Screw/pin composition	Drill diameter (mm)	Tap diameter (mm)	Screw/pin diameter (mm)	Screw/pin length (mm)	Plate length (mm)	Plate width (mm)	Plate thickness (mm)
**Titanium osteosynthesis systems**										
CrossDrive 1.5 mm (2006)	KLS Martin Group (Gebrüder Martin GmbH & Co., Tuttlingen, Germany)	100% titanium (by stamping)	90% titanium 6% aluminium 4% vanadium (Ti6Al4V)	1.1	None	1.5	6.0	18.5	3.5	0.6
CrossDrive 2.0 mm (2006)				1.5	None	2.0	6.0	25.5	5.0	1.0
CrossDrive 1.5 mm (2018)		100% titanium (by milling)		1.1	None	1.5	6.0	18.5	3.5	0.6
CrossDrive 2.0 mm (2018)				1.5	None	2.0	6.0	25.5	5.0	1.0
MaxDrive 1.5mm^a^			90% titanium 6% aluminium 4% vanadium (Ti6Al4V)^a^	1.1	None	1.5	6.0	18.5	3.5	0.6
MaxDrive 2.0mm^a^				1.5	None	2.0	6.0	25.5	5.0	1.0
**Biodegradable osteosynthesis systems**										
Inion CPS 2.0 mm	Inion Oy (Tampere, Finland)	70–78.5% PLLA 16–24% PDLLA 4.5–6% TMC^b^	70–78.5% PLLA 16–24% PDLLA 4.5–6% TMC^b^	1.75	2.0	2.0	7.0	28.0	7.0	1.3
Inion CPS 2.5 mm				2.25	2.5	2.5	6.0	32.0	8.5	1.6
LactoSorb 2.0 mm	Biomet Microfixation (Jacksonville, Florida)	82% PLLA 18% PGA	82% PLLA 18% PGA	1.7	2.0	2.0	7.0	28.5	7.0	1.3
Macropore 2.0 mm	Medtronic, Inc. (Minneapolis, USA)	70% PLLA 30% PDLLA	70% PLLA 30% PDLLA	1.5	2.0	2.0	6.0	25.0	6.7	1.2
Polymax 2.0 mm	Mathys Medical Ltd. (Bettlach Switzerland)	70% PLLA 30% PDLLA	70% PLLA 30% PDLLA	2.0^c^		2.0	6.0	28.0	6.0	1.3
BioSorb FX 2.0 mm	ConMed Linvatec Biomaterials Ltd. (Tampere, Finland)	SR 70% PLLA SR 30% PDLLA	SR 70% PLLA SR 30% PDLLA	1.5	2.0	2.0	6.0	25.5	5.5	1.3
ResorbX 2.1 mm	KLS Martin Group (Gebrüder Martin GmbH & Co., Tuttlingen, Germany)	100% PDLLA	100% PDLLA	1.8	2.1	2.1	7.0	26.0	6.0	1.0
SonicWeld Rx + SonicPins Rx (Rx/Rx) 2.1mm^d^		100% PDLLA	100% PDLLA (pin)	1.6	None or 2.0	2.1	7.0	26.0	6.0	1.0
SonicWeld xG + SonicPins Rx (xG/Rx) 2.1mm^d^		85% PLLA 15% PGA	100% PDLLA (pin)	1.6	None or 2.0	2.1	7.0	26.0	6.0	1.0
SonicWeld xG + SonicPins xG (xG/xG) 2.1mm^d^		85% PLLA 15% PGA	85% PLLA 15% PGA (pin)	1.6	None or 2.0	2.1	7.0	26.0	6.0	1.0
Osteotrans-MX	Teijin Medical Technologies Co., Ltd. (Osaka, Japan)	60% PLLA 40% uHA	70% PLLA 30% uHA	1.6	2.0	2.0	8.0	28.0	4.5	1.0
Osteotrans-MX										1.4

*PLLA* poly-l-lactic acid, *PDLLA* poly-d,l-lactic acid, *TMC* trimethylene carbonate, *SR* self-reinforced, *PGA* poly-glycolic acid, *uHA* unsintered hydroxyapatite.

^a^The MaxDrive screws have an adjusted screw head, compared to the CrossDrive screws, to improve screw grip while the plates of corresponding MaxDrive and CrossDrive (2018) systems are identical.

^b^The manufacturer does not publicly report the exact composition of the copolymers.

^c^Self-drilling tap.

^d^These systems were tested without tapping (as instructed by the manufacturer) and with tapping the burr holes.

### Optimal tap, pull-out load and stiffness of SonicPins

Tapping the burr hole is not part of the manufacturer’s standard application method for SonicPins. However, we hypothesized that tapping the burr hole whenever applied in cortical bone only can increase the axial pull-out load by increasing the contact area and mechanical retention of the fusioned pin in the cortical bone layer. Therefore, a pilot study was conducted to determine the optimal tap diameter of SonicPins Rx in a cortical bone model. We preferred fine threaded taps over coarse threaded taps for this pilot study because fine threads increase the surface contact of the pins with the bone segments more and are tapped more easily in hard materials (i.e., bone) compared to coarse ones. Thus, four different application methods were tested, viz., (1) the method prescribed by the manufacturer, i.e., 1.6 mm diameter drill without tapping the burr hole; (2) tapping after drilling the burr hole (i.e., 1.6 mm diameter drill) with 1.7 × 0.20; (3) 1.8 × 0.20; and (4) 2.0 × 0.25 mm taps (diameter x pitch of taps in mm; all fine threaded taps) to increase the contact area of the pins with the smooth cortical burr holes.

**Figure 1:**
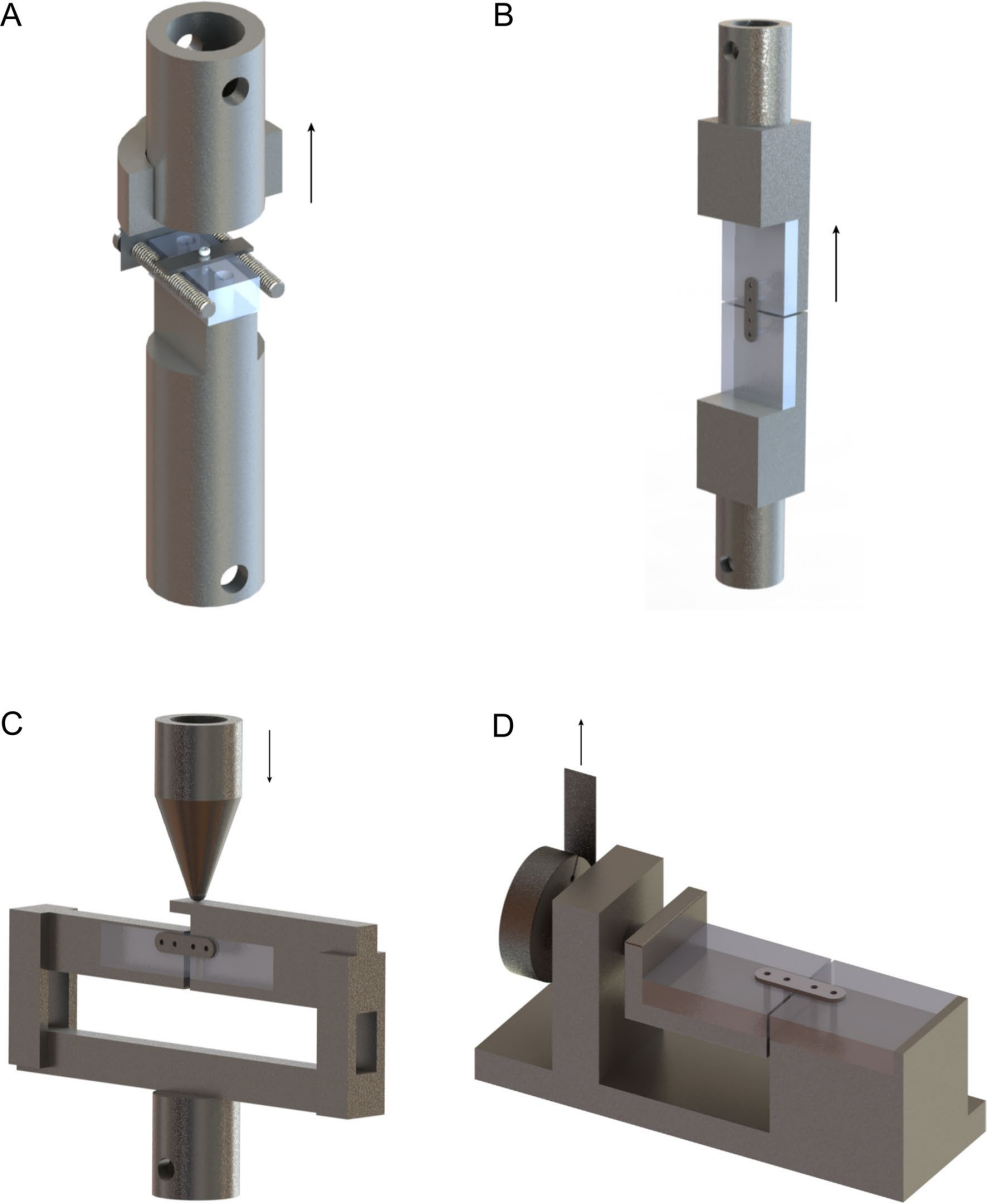
The pull-out **(a)**, tensile **(b)**, side bending **(c)**, and torsion **(d)** test setups. The arrows indicate the direction of the applied force.

The pull-out tests simulated the relatively high axial pull-out forces of in vivo situations (e.g., cranial reconstructions). Polymethylmethacrylate (PMMA) blocks (30.0 × 15.0 × 6.0 mm) were used to simulate bone segments^22–24^. The burr holes were drilled perpendicular to the surface of the PMMA block using the prescribed drill (i.e., 1.6 mm diameter) with water cooling. After drilling and tapping/not tapping the burr hole, the burr holes were irrigated with saline to simulate in situ lubrication. A titanium plate (25.0 × 6.0 × 1.0 mm) with a single 2.3 mm hole was placed above the burr hole and the SonicPins Rx were applied, as prescribed by the manufacturer, by a single researcher (BG; Fig. 1a). The titanium plate was chosen in order to ensure that the forces were transferred directly to the pins. The thickness of the titanium plate of 1.0 mm was specifically chosen as the osteosynthesis plates corresponding to these SonicPins have the same thickness of 1.0 mm. Therefore, the test setup did not interfere with the length of the screw in the bone compared to the in vivo situation. The PMMA-blocks with the SonicPins Rx in situ were stored for 24 h in a water tank containing 37.1 °C water to simulate SonicPins Rx relaxation at body temperature. Saline was avoided to prevent possible corrosion of the test environment. The use of water instead of saline was not expected to influence the test results^12,18^. Subsequently, the tests were performed in another tank, containing water of the same temperature, mounted on the test machine (Zwick/Roell TC-FR2, 5TS.D09, 2.5 kN Test machine; force accuracy 0.2%, positioning accuracy 0.0001 mm; Zwick/Roell Nederland, Venlo, The Netherlands). All the samples were analysed in the same test machine using a standardized protocol (see the Mechanical tests and Statistical analyses described below).

The SonicPins xG pins were applied using both the method prescribed by the manufacturer (i.e., without tapping the burr hole) and with a tap that yielded the highest pull-out load in the abovementioned pilot study. Subsequently, both were subjected to the pull-out tests described above.

### Torque, tensile, side bending and torsion tests

All the selected osteosynthesis systems, consisting of straight four-hole plates with intermediate spaces, underwent three different mechanical tests, i.e. tensile, three-point side bending, and torsion tests. The tensile test was a standard loading test since an osteosynthesis system is unavoidably exposed to these forces after adequate repositioning of the bone segments^25,26^. The three-point side bending tests simulated the bending forces a mandible is exposed to, e.g., after a bilateral sagittal split osteotomy (BSSO)^27^. The torsion test simulated the high torsion forces seen with, e.g., a fracture of the mandibular symphysis^28^.

Once again, PMMA blocks were used to simulate bone segments. Two 40.0 × 36.0 × 6.0 mm blocks were used for the tensile and torsion tests, while two 40.0 × 15.0 × 6.0 mm blocks were used for the side bending test (Fig. 1b-d). The size of the side bending test blocks was different to avoid premature contact of the PMMA-blocks during testing. The burr holes were drilled perpendicular to the surface of the PMMA block using the prescribed drills with cooling (Table 1). After drilling and, optionally, tapping, the burr holes were irrigated with saline to simulate in situ lubrication. All three SonicWeld systems were also tested using the preferred tap as determined in the abovementioned pilot study. The two PMMA-blocks were fixated using an osteosynthesis system without interfragmentary contact between the PMMA-blocks to simulate the most unfavourable situation^29^. All the osteosynthesis systems were applied according to the manufacturers’ instructions, with two screws or pins in each PMMA-block (in total 4 screws/pins per plate, two at each side of the fracture; Fig. 1b-d) and by the same researcher (BG).

The osteosynthesis screws were inserted with the prescribed screw drivers, and using the mean applied torque, by the same four experienced OMF-surgeons (RRMB, FKLS, GMR, and JJ) defined in a previous study^30^. Since the SonicWeld systems use ultra-sound activated SonicPins instead of screws, no torque could be applied or measured. To standardize the application of these pins, we used a minimum of 1 s and a maximum of 2 s to insert each SonicPin. A fixed time was not chosen as the time needed to melt each pin varies slightly, similar to the clinical situation, and the surgeon will melt the pin until it is correctly applied. Since the MaxDrive (i.e., 1.5 and 2.0 mm) and Osteotrans-MX systems had not been developed yet when doing the previous study^30^, the same four experienced OMF-surgeons (RRMB, FKLS, GMR, and JJ)^30^ were asked to participate in this study and to insert the 6 screws of both the MaxDrive and Osteotrans-MX systems into the same standardized, pre-drilled PMMA-blocks (36.0 × 36.0 × 6 mm) as they would do in the clinic (i.e., ‘hand tight’). The test setup and conditions to assess the applied torque were identical to that described in the previous study^30^. Additionally, like the previous study, one researcher (BG) inserted the 6 screws of both systems until fracture occurred (i.e., torque needed for screw breakage). The torque was recorded using a torque measurement meter (Nemesis Howards Torque Gauge, Smart MT-TH 50 sensor, accuracy 2.5 Nmm, range 0–680 Nmm).

The PMMA-blocks with the osteosynthesis systems in situ were stored for 24 h in a tank containing water at 37.1 °C to simulate relaxation of the systems at body temperature. Subsequently, the tests were performed in another tank containing water with the same temperature. All the samples were tested in the same test machine and analysed using a standardized protocol (see “Mechanical tests” and “Statistical analysis” below).

### Mechanical tests

All the mechanical tests were performed with the same machine by the same researcher (CCR). In the pull-out test, the SonicPins were subjected to axial forces with a constant speed of 5 mm/min until the SonicPins were pulled out or fractured (Fig. 1a)^21^. During the tensile tests, the osteosynthesis systems were subjected to tensile forces with a constant speed of 5 mm/min until fracture of the plate or screws/pins occurred (Fig. 1b). In the side bending tests, the PMMA-blocks were fixated at both ends and the osteosynthesis plate was loaded in the centre with a constant speed of 30 mm/min until the plate bent by 30° (Fig. 1c). The torsion test consisted of rotating the two PMMA-blocks along the long axis with a constant speed of 90°/min until 160° torsion of the plate occurred (Fig. 1d).

The applied force and displacement were measured with a frequency of 500 Hz. These results were presented as a force–displacement graph. The pull-out and tensile tests yielded a maximum load (in N) and stiffness (in N/mm). The outcome measures for the side bending and torsion tests were stiffness (in N/mm) and torsional stiffness (Nmm/°rotation), respectively. The stiffness of the pull-out, tensile, and side bending tests were determined using the force–displacement graph. Herein, the direction coefficient of the line connecting the points of the 25% and 75% maximum force in the elastic region was determined. This excluded inaccuracies at the beginning and end of the force–displacement graphs. The torsional stiffness was calculated using the following formulas:

1. T = F x r
2. *k* = T/Φ

where T is the torque (Nmm), F is the force (N), r is the radius (20 mm in this test setup), *k* is the torsional stiffness (Nmm/° rotation), and Φ is the angle of twist (°). The origin of failure of all tests was recorded.

In this study, all 15 biodegradable and six titanium osteosynthesis systems were mechanically tested. Of these, seven biodegradable and two titanium systems had been tested in a previous study by the author’s research group^12,18^. The test setups and environment used in the previous and current study were identical. To ensure a correct direct comparison, a biodegradable system that was tested in the previous study and that had not been altered by the manufacturer over time (i.e., KLS SonicWeld Rx_plate_/Rx_pins_ 2.1 mm osteosynthesis system) was tested again in all three of the current study’s test setups. The tensile load and tensile, side bending, and torsional stiffness were statistically compared and the force–displacement graphs were visually inspected. Direct comparability of all the mechanical tests was considered appropriate whenever the previous and current studies’ outcome values did not differ statistically and the force–displacement graphs were similar.

### Statistical analysis

The assumption of normal distribution of data was tested by visually examining the Q-Q plots and the Shapiro–Wilk test. All the data were presented as means with standard deviations (SD). The Levene’s test was performed to check the assumption of equality of variances of data. The mean pull-out and tensile load, and pull-out, tensile, side bending and torsional stiffness of the included osteosynthesis systems were statistically compared using a one-way analysis of variance (ANOVA). To correct for multiple testing, the Tukey’s or Dunett’s T3 post hoc test was performed in case of the assumption of equal or unequal variances, respectively. P-values less than 0.05 (two-tailed) were considered statistically significant. All the analyses were performed in Statistical Package of Social Sciences (SPSS) 23 (IBM SPSS Statistics for Windows, Version 23.0. Armonk, NY: IBM Corp.).

## Results

### Optimal tap and pull-out load of SonicPins

**Figure 2:**
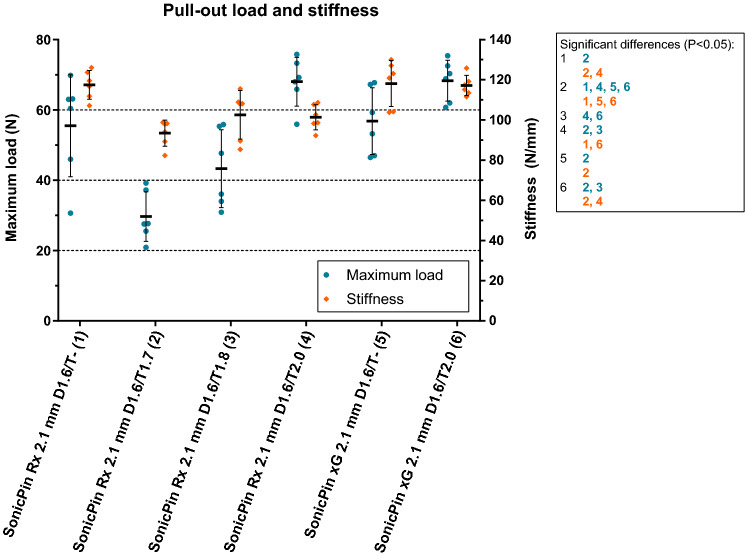
The pull-out load and stiffness of SonicPins Rx and xG. The characters in blue and orange represent significant differences in maximum load (N) and stiffness (N/mm). *D, *drill diameter (mm)*; T, *tap diameter (mm)*. Error bars: *mean values ± standard deviation. All the load and stiffness values, including the P-values of the pairwise comparisons, are reported in Supplementary Table S1.

The mean pull-out load and stiffness of the SonicPins Rx without tap and with 1.7, 1.8, and 2.0 mm taps are presented in Fig. 2 and Supplementary Table S1. The SonicPins Rx with a tap diameter of 2.0 mm had the highest mean pull-out load compared to those with 1.7 and 1.8 mm diameter taps. Therefore, the SonicPins xG were also subjected to the pull-out test without and with tapping the burr hole with a 2.0 mm diameter tap.

The pull-out load of the SonicPins Rx and SonicPins xG, both without tapping the burr hole, did not differ significantly (P > 0.999). Tapping the burr holes with a 2.0 mm tap did not improve the pull-out loads of the SonicPins Rx and xG compared to not tapping the burr holes (Rx: 68.0 (6.94) N vs. 55.5 (14.5) N, P = 0.474; xG: 68.3 (5.83) N vs. 56.8 (9.50) N, P = 0.712; Fig. 2 and Supplementary Table S1). Tapping the burr hole with a 1.7 mm tap (29.7 (7.08) N) resulted in significantly lower pull-out load compared to not tapping the SonicPins Rx burr hole (P = 0.001), whereas there were no significant differences in pull-out load between tapping with a 1.8 mm tap (42.5 (11.1) N) and not tapping the burr hole (P = 0.474).

The pull-out stiffness of the SonicPin Rx (117 (7.14) N/mm) compared to the SonicPin xG (118 (11.4) N/mm), both without tapping the burr hole, did not differ significantly (P > 0.999; Fig. 2 and Supplementary Table S1). Tapping the SonicPins Rx burr hole with 2.0 mm tap significantly lowered the pull-out stiffness compared to not tapping the burr hole (101 (6.25) N/mm vs. 117 (7.14) N/mm; P = 0.024). The pull-out stiffness after tapping the SonicPins xG burr hole with a 2.0 mm tap was not significantly different compared to not tapping the burr hole (117 (5.05) N/mm vs. 118 (11.4) N/mm; P > 0.999).

The shaft of four of the six SonicWeld Rx pins subjected to a 1.7 mm tap failed whereas the heads of all the other SonicPins Rx and xG pins failed.

### Torque of osteosynthesis screws

The mean torque applied to the KLS MaxDrive 1.5 and 2.0 mm screws by four experienced OMF-surgeons (i.e., ‘hand tight’) was 319 (65.3) and 407 (138) Nmm, respectively (Supplementary Table S2 and Fig. S4). The mean torque applied to the same systems until screw breakage was 528 (16.9) and > 680 Nmm (i.e., maximum of torque meter range achieved), respectively. Comparatively, the applied hand-tight torque of the MaxDrive 1.5 mm screws were significantly higher than the CrossDrive 1.5 mm screws (P = 0.046), while the torque applied to the MaxDrive 2.0 mm screws did not differ significantly with the CrossDrive 2.0 mm screws (P > 0.999). All the Osteotrans-MX 2.0 mm screw heads failed during insertion in PMMA by the OMF-surgeons, before the screws were fully in. Therefore, these osteosynthesis systems could not be tested in the setups. The mean torque applied to all the other included osteosynthesis systems (i.e., ‘hand tight’ and until screw breakage), as well as the statistical comparisons, are presented in Supplementary Table S2 and Fig. S4. The mean torque applied to all the titanium screws with both the ‘hand tight’ and ‘breakage’ method was significantly higher than that applied to the biodegradable screws (Supplementary Table S2 and Fig. S4).

### Tensile, side bending and torsion tests

Firstly, to test the assumption that our previous and current studies’ set-ups were identical^12,18^, the KLS SonicWeld Rx/Rx 2.1 mm system was tested and compared to the results of the same system derived from our previous study^12^. The curves of the previous and current force–displacement graphs (i.e., tensile, side bending and torsion tests) were similar (Supplementary Fig. S2-4). The results of the mean tensile load (previous: 115 (8.69) vs. current: 112 (2.25) N; P = 0.511) and stiffness (495 (34.0) vs. 489 (21.9) N/mm; P = 0.718), and side bending (1.11 (0.09) vs. 1.08 (0.08) N/mm; P = 0.656) and torsion stiffness (2.13 (0.30) vs. 2.12 (0.26) Nmm/°; P = 0.932) did not differ significantly. Therefore, direct comparison of the previously and currently tested osteosynthesis systems was considered appropriate for the rest of this study.

**Figure 3:**
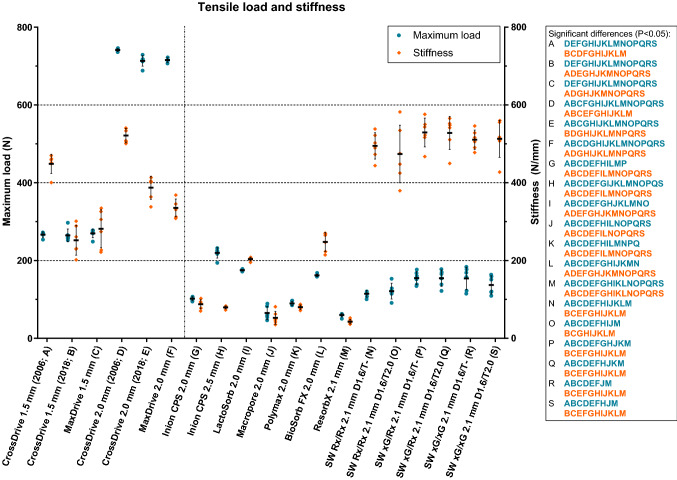
The tensile load and stiffness of all the included osteosynthesis systems. The characters in blue and orange represent significant differences in maximum load (N) and stiffness (N/mm). *D, *drill diameter (mm);* T, *tap diameter (mm).* Error bars: *mean values ± standard deviation. The dotted line separates the titanium (left) and biodegradable systems (right). All the load and stiffness values, including the P-values of the pairwise comparisons, are reported in Table 2.

The torque applied to the osteosynthesis screws for the tensile tests corresponded to the mean torque applied by the four experienced OMF-surgeons (Table 2). The mean tensile load and stiffness of all the systems, including statistical comparisons, are presented in Fig. 3 and Table 2. The tensile loads of all the titanium systems were significantly higher compared to the biodegradable systems. The tensile loads of the CrossDrive (2006 and 2018) and MaxDrive systems were similar. However, the tensile stiffness of the CrossDrive (2018) and MaxDrive 1.5 mm were significantly lower than the CrossDrive (2006) 1.5 mm system (P < 0.001 and P = 0.007, respectively). The displacement until fracture occured (i.e., in the force–displacement graph) of the CrossDrive (2018) and MaxDrive systems was significantly higher (2.11 (0.23) and 1.83 (0.11) mm, respectively) than that of the CrossDrive (2006) system (1.12 (0.07) mm; both P < 0.001; Supplementary Fig. S5). Similary, the stiffness of the CrossDrive (2018) and MaxDrive 2.0 mm was significantly lower compared to the CrossDrive (2006) 2.0 mm system (P = 0.001 and P < 0.001, respectively) and the displacement until fracture occurred was higher in the former two systems (3.05 (0.08) and 3.37 (0.10) mm, respectively) compared to the latter system (2.42 (0.11) mm; both P < 0.001; Supplementary Fig. S6). The higher displacement until fracture of the CrossDrive (2018) and MaxDrive systems indicates higher ductility than the CrossDrive (2006) systems (Supplementary Fig. S5 and S6). Furthermore, the tensile stiffness of the SonicWeld Rx and xG systems, regardless of the method used (i.e., without or with tapping the burr hole), was significantly higher than the other biodegradable systems (Fig. 3 and Table 2). It was noted that the tensile load and stiffness of the SonicWeld Rx and xG systems were significantly higher than the Resorb X system (i.e., a system with the same composition and dimensions, but with screws instead of SonicPins). There were no significant differences in tensile load and stiffness between the SonicWeld Rx and xG systems. The Inion CPS 2.5 mm system’s tensile load was the highest among all the biodegradable systems. The origin of the titanium and SonicWeld systems’ failure during the tensile test was plate breakage while all the other biodegradable systems experienced screw-head shearing.

**Table 2:** The tensile load and stiffness of all the included osteosynthesis systems.

Ref	System	Mean torque applied to screws (SD) in Nmm	Mean Fmax (SD) in N	P-values (pairwise comparison)	Mean stiffness (SD) in N/mm	P-values (pairwise comparison)
A	CrossDrive 1.5 mm (2006)	251 (1.54)	267 (6.74)	B: > 0.999; C: > 0.999; **D: < 0.001; E: < 0.001; F: < 0.001; G: < 0.001; H: 0.006; I: < 0.001; J: < 0.001; K: < 0.001; L: < 0.001; M: < 0.001; N: < 0.001; O: < 0.001; P: < 0.001; Q: 0.001; R: 0.006; S: 0.001**	449 (24.7)	**B: < 0.001; C: 0.007; D: 0.020;** E: 0.177; F: **0.001; G: < 0.001; H: < 0.001; I: < 0.001; J: < 0.001; K: < 0.001; L: < 0.001; M: < 0.001;** N: 0.679; O: > 0.999; P: 0.107; Q: 0.202; R: 0.104; S: 0.563
B	CrossDrive 1.5 mm (2018)	247 (0.53)	265 (16.4)	A: > 0.999; C: > 0.999; **D: < 0.001; E: < 0.001; F: < 0.001; G: < 0.001; H: 0.037; I: 0.001; J: < 0.001; K: < 0.001; L: 0.001; M: < 0.001; N: < 0.001; O: < 0.001; ; P: < 0.001; Q: < 0.001; R: 0.003; S: < 0.001**	252 (38.3)	**A: < 0.001;** C: > 0.999; **D: < 0.001; E: 0.005;** F: 0.101; **G: 0.003; H: 0.004;** I: 0.527; **J: 0.001; K: 0.004;** L: > 0.999; **M: 0.001; N: < 0.001; O: 0.016; P: < 0.001; Q: < 0.001; R: < 0.001; S: < 0.001**
C	MaxDrive 1.5 mm	320 (0.48)	270 (10.9)	A: > 0.999; B: > 0.999; **D: < 0.001; E: < 0.001; F: < 0.001; G: < 0.001; H: 0.004; I: < 0.001; J: < 0.001; K: < 0.001; L: < 0.001; M: < 0.001; N: < 0.001; O: < 0.001; P: < 0.001; Q: < 0.001; R: 0.004; S: < 0.001**	283 (49.0)	**A: 0.007;** B: > 0.999; **D: 0.001;** E: 0.099; F: 0.794; **G: 0.006; H: 0.006;** I: 0.289; **J: 0.002; K: 0.006;** L: 0.998; **M: 0.003; N: 0.001; O: 0.039; P: < 0.001; Q: < 0.001;R: 0.001; S: 0.001**
D	CrossDrive 2.0 mm (2006)	370 (1.09)	741 (4.08)	**A: < 0.001; B: < 0.001; C: < 0.001;** E: 0.108**; F: 0.001; G: < 0.001; H: < 0.001; I: < 0.001; J: < 0.001; K: < 0.001; L: < 0.001; M: < 0.001; N: < 0.001; O: < 0.001; P: < 0.001; Q: < 0.001; R: < 0.001; S: < 0.001**	521 (18.6)	**A: 0.020; B: < 0.001; C: 0.001; E: 0.001; F: < 0.001; G: < 0.001; H: < 0.001; I: < 0.001; J: < 0.001; K: < 0.001; L: < 0.001; M: < 0.001;** N: 0.992; O: 0.995; P: > 0.999; Q: > 0.999; R: > 0.999; S: > 0.999
E	CrossDrive 2.0 mm (2018)	368 (1.22)	713 (13.5)	**A: < 0.001; B: < 0.001; C: < 0.001;** D: 0.108; F: > 0.999**; G: < 0.001; H: < 0.001; I: < 0.001; J: < 0.001; K: < 0.001; L: < 0.001; M: < 0.001; N: < 0.001; O: < 0.001; P: < 0.001; Q: < 0.001; R: < 0.001; S: < 0.001**	387 (29.5)	A: 0.177; **B: 0.005;** C: 0.099; **D: 0.001;** F: 0.326**; G: < 0.001; H: < 0.001; I: 0.001; J: < 0.001; K: < 0.001; L: < 0.001; M: < 0.001; N: 0.014;** O: 0.674; **P: 0.003; Q: 0.008; R: 0.002; S: 0.033**
F	MaxDrive 2.0 mm	408 (0.34)	716 (5.91)	**A: < 0.001; B: < 0.001; C: < 0.001; D: 0.001;** E: > 0.999; **G: < 0.001; H: < 0.001; I: < 0.001; J: < 0.001; K: < 0.001; L: < 0.001; M: < 0.001; N: < 0.001; O: < 0.001; P: < 0.001; Q: < 0.001; R: < 0.001; S: < 0.001**	335 (22.8)	**A: 0.001;** B: 0.101; C: 0.794; **D: < 0.001;** E: 0.326**; G: < 0.001; H: < 0.001; I: 0.001; J: < 0.001; K: < 0.001 L: 0.007; M: < 0.001; N: 0.001;** O: 0.169; **P: < 0.001; Q: 0.001; R: < 0.001; S: 0.004**
G	Inion CPS 2.0 mm	74.3 (0.31)	102 (5.11)	**A: < 0.001; B: < 0.001; C: < 0.001; D: < 0.001; E: < 0.001; F: < 0.001; H: < 0.001; I: < 0.001;** J: 0.091; K: 0.162; **L: < 0.001; M: < 0.001;** N: 0.504; O: 0.847; **P: 0.015;** Q: 0.052; R: 0.193; S: 0.355	87.6 (11.7)	**A: < 0.001; B: 0.003; C: 0.006; D: < 0.001; E: < 0.001; F: < 0.001;** H: 0.992; **I: < 0.001;** J: 0.140; K: 0.999; **L: < 0.001; M: 0.004; ;N: < 0.001; O: 0.002; P: < 0.001; Q: < 0.001; R: < 0.001; S: < 0.001**
H	Inion CPS 2.5 mm	157 (0.77)	220 (13.4)	**A: 0.006; B: 0.037; C: 0.004; D: < 0.001; E: < 0.001; F: < 0.001; G: < 0.001; I: 0.015; J: < 0.001; K: < 0.001; L: 0.004; M: < 0.001; N: < 0.001; O: < 0.001; P: 0.003; Q: 0.013;** R: 0.074; **S: 0.005**	79.5 (3.74)	**A: < 0.001; B: 0.004; C: 0.006; D: < 0.001; E: < 0.001; F: < 0.001;** G: 0.992; **I: < 0.001;** J: 0.284; K: > 0.999; **L: < 0.001; M: < 0.001; N: < 0.001; O: 0.002; P: < 0.001; Q: < 0.001; R: < 0.001; S: < 0.001**
I	LactoSorb 2.0 mm	98.0 (0.48)	175 (2.40)	**A: < 0.001; B: 0.001; C: < 0.001; D: < 0.001; E: < 0.001; F: < 0.001; G: < 0.001; H: 0.015; J: 0.001; K: < 0.001; L: 0.002; M: < 0.001; N: < 0.001; O: 0.042;** P: 0.562; Q: 0.803; R: 0.969; S: 0.271	208 (4.82)	**A: < 0.001;** B: 0.527; C: 0.289; **D: < 0.001; E: 0.001; F: 0.001; G: < 0.001; H: < 0.001; J: < 0.001; K: < 0.001;** L: 0.186; **M: < 0.001; N: < 0.001; O: 0.011; P: < 0.001; Q: < 0.001; R: < 0.001; S: 0.001**
J	Macropore 2.0 mm	62.4 (0.47)	65.1 (16.9)	**A: < 0.001; B: < 0.001; C: < 0.001; D: < 0.001; E: < 0.001; F: < 0.001;** G: 0.091; **H: < 0.001; I: 0.001;** K: 0.400; **L: 0.001;** M: > 0.999; **N: 0.019; O: 0.035; P: < 0.001; Q: 0.001; R: 0.013; S: 0.014**	52.9 (16.6)	**A: < 0.001; B: 0.001; C: 0.002; D: < 0.001; E: < 0.001; F: < 0.001;** G: 0.140; H: 0.284; **I: < 0.001;** K: 0.276; **L: < 0.001;** M: 0.999; **N: < 0.001; O: 0.001; P: < 0.001; Q: < 0.001; R: < 0.001; S: < 0.001**
K	Polymax 2.0 mm	57.1 (0.58)	89.7 (5.53)	**A: < 0.001; B: < 0.001; C: < 0.001 ; D: < 0.001; E: < 0.001; F: < 0.001;** G: 0.162; **H: < 0.001; I: < 0.001;** J: 0.400; **L: < 0.001; M: < 0.001; N: 0.021;** O: 0.314; **P: 0.005; Q: 0.019;** R: 0.086; S: 0.127	80.1 (5.74)	**A: < 0.001; B: 0.004; C: 0.006; D: < 0.001; E: < 0.001; F: < 0.001;** G: 0.999; H: > 0.999; **I: < 0.001;** J: 0.276; **L: < 0.001; M: < 0.001; N: < 0.001; O: 0.002; P: < 0.001; ;Q: < 0.001 ;R: < 0.001; S: < 0.001**
L	BioSorb FX 2.0 mm	81.2 (0.41)	162 (3.18)	**A: < 0.001; B: 0.001; C: < 0.001; D: < 0.001; E: < 0.001; F: < 0.001; G: < 0.001; H: 0.004; I: 0.002; J: 0.001; K: < 0.001; M: < 0.001; N: 0.001;** O: 0.130; P: > 0.999; Q: > 0.999; R: > 0.999; S: > 0.999	248 (24.3)	**A: < 0.001;** B: > 0.999; C: 0.998; **D: < 0.001; E: < 0.001; F: 0.007; G: < 0.001; H: < 0.001;** I: 0.186; **J: < 0.001; K: < 0.001; M: < 0.001; N: < 0.001; O: 0.018; P: < 0.001; Q: < 0.001; R: < 0.001; S: < 0.001**
M	Resorb X 2.1 mm	56.1 (0.23)	59.9 (4.73)	**A: < 0.001; B: < 0.001; C: < 0.001; D: < 0.001; E: < 0.001; F: < 0.001; G: < 0.001; H: < 0.001; I: < 0.001;** J: > 0.999; **K: < 0.001; L: < 0.001; N: < 0.001; O: 0.021; P: 0.001; Q: 0.003; R: 0.017; S: 0.015**	42.9 (5.82)	**A: < 0.001; B: 0.001; C: 0.003; D: < 0.001; E: < 0.001; F: < 0.001; G: 0.004; H: < 0.001; I: < 0.001;** J: 0.999; **K: < 0.001; L: < 0.001; N: < 0.001; O: 0.001; P: < 0.001; Q: < 0.001; R: < 0.001; S: < 0.001**
N	SW Rx/Rx 2.1 mm (D1.6/T-)	NA	115 (8.69)	**A: < 0.001; B: < 0.001; C: < 0.001; D: < 0.001; E: < 0.001; F: < 0.001;** G: 0.504; **H: < 0.001; I: < 0.001; J: 0.019; K: 0.021; L: 0.001; M: < 0.001;** O: > 0.999; P: 0.053; Q: 0.164; R: 0.465; S: 0.879	495 (34.0)	A: 0.679; **B: < 0.001;** C: **0.001;** D: 0.992; **E: 0.014; F: 0.001; G: < 0.001; H: < 0.001; I: < 0.001; J: < 0.001; K: < 0.001; L: < 0.001; M: < 0.001;** O: > 0.999; P: 0.995; ;Q: 0.999; R: > 0.999; S: > 0.999
O	SW Rx/Rx 2.1 mm (D1.6/T2.0)	NA	121 (20.2)	**A: < 0.001; B: < 0.001; C: < 0.001;** **D: < 0.001; E: < 0.001; F: < 0.001;** G: 0.847; **H: < 0.001; I: 0.042; J: 0.035;** K: 0.314; L: 0.130; **M: 0.021;** N: > 0.999; P: 0.453; Q: 0.629; R: 0.874; S: > 0.999	529 (37.0)	A: > 0.999; **B: 0.016; C: 0.039;** D: 0.995; E: 0.674; F: 0.169; **G: 0.002; H: 0.002; I: 0.011; J: 0.001; K: 0.002; L: 0.018; M: 0.001;** N: > 0.999; P: 0.993; Q: 0.997; R: > 0.999; S: > 0.999
P	SW xG/Rx 2.1 mm (D1.6/T-)	NA	155 (16.6)	**A: < 0.001; B: < 0.001; C: < 0.001; D: < 0.001; E: < 0.001; F: < 0.001; G: 0.015; H: 0.003;** I: 0.562; **J: < 0.001; K: 0.005;** L: > 0.999; **M: 0.001;** N: 0.053; O: 0.453; Q: > 0.999; R: > 0.999; S: 0.999	529 (37.0)	A: 0.107; **B: < 0.001; C: < 0.001;** D: > 0.999; **E: 0.003; F: < 0.001; G: < 0.001; H: < 0.001; I: < 0.001; J: < 0.001; K: < 0.001; L: < 0.001; M: < 0.001;** N: 0.995; O: 0.993; Q: > 0.999; R: > 0.999; S: > 0.999
Q	SW xG/Rx 2.1 mm (D1.6/T2.0)	NA	155 (21.1)	**A: 0.001; B: < 0.001; C: < 0.001; D: < 0.001; E: < 0.001; F: < 0.001;** G: 0.052; **H: 0.013;** I: 0.803; **J: 0.001; K: 0.019;** L: > 0.999; **M: 0.003;** N: 0.164; O: 0.629; P: > 0.999; R: > 0.999; S: > 0.999	528 (42.5)	A: 0.202; **B: < 0.001; C: < 0.001;** D: > 0.999; **E: 0.008; F: 0.001; G: < 0.001; H: < 0.001; I: < 0.001; J: < 0.001; K: < 0.001; L: < 0.001; M: < 0.001;** N: 0.999; O: 0.997; P: > 0.999; R: > 0.999; S: > 0.999
R	SW xG/xG 2.1 mm (D1.6/T-)	NA	154 (28.9)	**A: 0.006; B: 0.003; C: 0.004; D: < 0.001; E: < 0.001; F: < 0.001;** G: 0.193; H: 0.074; I: 0.969; **J: 0.013** K: 0.086; L: > 0.999; **M: 0.017;** N: 0.465; O: 0.874; P: > 0.999; Q: > 0.999; S: > 0.999	511 (24.9)	A: 0.104; **B: < 0.001; C: 0.001;** D: > 0.999; **E: 0.002; F: < 0.001; G: < 0.001; H: < 0.001; I: < 0.001; J: < 0.001; K: < 0.001; L: < 0.001; M: < 0.001;** N: > 0.999; O: > 0.999; P: > 0.999; Q: > 0.999; S: > 0.999
S	SW xG/xG 2.1 mm (D1.6/T2.0)	NA	137 (23.5)	**A: 0.001; B: < 0.001; C: < 0.001; D: < 0.001; E: < 0.001; F: < 0.001;** G: 0.355; **H: 0.005;** I: 0.271; **J: 0.014;** K: 0.127; L: 0.723; **M: 0.015;** N: 0.879; O: > 0.999; P: 0.999; Q: > 0.999; R: > 0.999	513 (47.8)	A: 0.563; **B: < 0.001; C: 0.001;** D: > 0.999; **E: 0.033**; **F: 0.004; G: < 0.001; H: < 0.001; I: 0.001; J: < 0.001; K: < 0.001; L: < 0.001; M: < 0.001;** N: > 0.999; O: > 0.999; P: > 0.999; Q: > 0.999; R: > 0.999;

*Ref* reference, also used in the pairwise comparisons column and in Fig. 3; *SD* standard deviation, *NA* not applicable.

The bold P-values represent the statistically significant values after correcting for multiple testing (P < 0.05).

**Figure 4:**
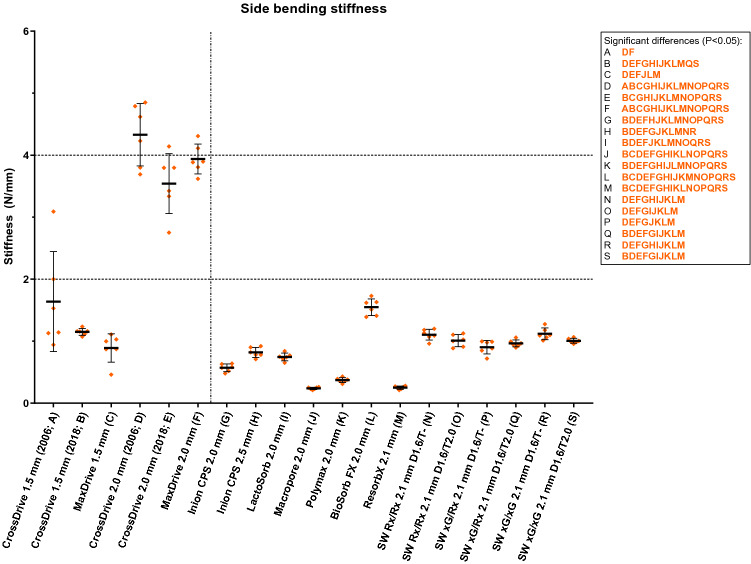
The side bending stiffness of all the included osteosynthesis systems. The characters in orange represent significant differences in stiffness (N/mm). *D, *drill diameter (mm)*; T, *tap diameter (mm). *Error bars: *mean values ± standard deviation (N/mm). The dotted line separates the titanium (left) and biodegradable systems (right). All the stiffness values, including the P-values of the pairwise comparisons, are reported in Table 3.

The torque applied to the osteosynthesis screws for the side bending and torsion tests corresponded to the mean torque applied by the experienced OMF-surgeons (Table 3). The side bending stiffness of the 1.5 mm titanium systems was comparable to the biodegradable systems (Fig. 4 and Table 3). The 2.0 mm titanium systems had significantly higher side bending stiffness compared to the 1.5 mm titanium and all the biodegradable systems. Of all the biodegradable systems, the BioSorb FX 2.0 mm system had the highest side bending stiffness (1.55 (0.13) N/mm). The side bending stiffness of all the included SonicWeld systems was significantly higher compared to the Resorb X system. None of the osteosynthesis systems fractured during the side bending tests.

**Table 3:** The side bending and torsional stiffness of all the included osteosynthesis systems.

Ref	System	Side-bending test			Torsion test		
		Mean torque applied to screws (SD) in Nmm	Mean stiffness (SD) in N/mm	P-values (pairwise comparison)	Mean torque applied to screws (SD) in Nmm	Mean torsional stiffness (SD) in Nmm/°	P-values (pairwise comparison)
A	CrossDrive 1.5 mm (2006)	248 (0.70)	1.64 (0.81)	B: 0.995; C: 0.877; **D: 0.007;** E: 0.064; **F: 0.026;** G: 0.468; H: 0.762; I: 0.670; J: 0.218; K: 0.297; L: > 0.999; M: 0.224; N: 0.988; O: 0.948; P: 0.862; Q: 0.914; R: 0.991; S: 0.943	249 (1.36)	8.92 (0.52)	**B: < 0.001; C: < 0.001; D: 0.002; E: 0.002; F: 0.002; G: < 0.001; H: < 0.001; I: < 0.001;** J: > 0.999; **K: < 0.001; L: 0.003; M: < 0.001; N: < 0.001; O: < 0.001; P: < 0.001; Q: < 0.001; R: < 0.001; S: < 0.001**
B	CrossDrive 1.5 mm (2018)	248 (1.43)	1.15 (0.05)	A: 0.995; ; C: 0.671; **D: 0.001; E: 0.002; F: < 0.001; G: < 0.001; H: 0.002; I: 0.001; J: < 0.001; K: < 0.001; L: 0.019; M: < 0.001;** N: > 0.999; O: 0.488; P: 0.075; **Q: 0.016;** R: > 0.999; **S: 0.037**	248 (0.30)	3.61 (0.41)	**A: < 0.001;** C: 0.999; **D: 0.001; E: 0.001; F: < 0.001;** G: 0.138; **H: < 0.001;** I: > 0.999; **J: 0.001; K: 0.002; L: 0.001; M: 0.005; N: 0.005;** O: 0.971; P: 0.291; Q: 0.398; **R: 0.036;** S: 0.654
C	MaxDrive 1.5 mm	320 (0.16)	0.89 (0.23)	A: 0.877; B: 0.671; **D: < 0.001; E: < 0.001; F: < 0.001;** G: 0.436; H: > 0.999; I: 0.996; **J: 0.033;** K: 0.082; **L: 0.020; M: 0.035;** N: 0.895; O: > 0.999; P: > 0.999; Q: > 0.999; R: 0.858; S: > 0.999	320 (0.44)	3.30 (0.30)	**A: < 0.001;** B: 0.999; **D: 0.001; E: 0.001; F: < 0.001; G: 0.007; H: < 0.001;** I: 0.666; **J: 0.001; K: 0.001; L: 0.001; M: 0.004; N: 0.004;** O: > 0.999; P: 0.725; Q: 0.901; R: 0.097; S: 0.933
D	CrossDrive 2.0 mm (2006)	370 (1.02)	4.33 (0.50)	**A: 0.007; B: 0.001; C: < 0.001;** E: 0.640; F: 0.987; **G: < 0.001; H: < 0.001; I: < 0.001; J: < 0.001; K: < 0.001; L: 0.001; M: < 0.001; N: 0.007; O: < 0.001; P: < 0.001; Q: 0.001; R: 0.001; S: 0.001**	368 (1.97)	27.8 (3.59)	**A: 0.002; B: 0.001; C: 0.001;** E: 0.860; F: 0.570; **G: 0.001; H: 0.013; I: 0.001; J: 0.001; K: 0.001** **L: 0.001; M: < 0.001; N: 0.002; O: < 0.001; P: < 0.001; Q: < 0.001; R: < 0.001; S: < 0.001**
E	CrossDrive 2.0 mm (2018)	369 (0.93)	3.54 (0.48)	A: 0.064; **B: 0.002; C: < 0.001;** D: 0.640; F: 0.979; **G: 0.001; H: 0.001; I: 0.001; J: 0.001; K: 0.001; L: 0.004; M: 0.001; N: 0.002; O: 0.001; P: 0.001; Q: 0.002; R: 0.002; S: 0.002**	369 (0.80)	23.4 (2.96)	**A: 0.002; B: 0.001; C: 0.001;** D: 0.860; F: > 0.999; G: **0.001;** H: **0.044;** I: **0.001;** J: **0.001;** K: **0.001;** L: **0.001;** M: < **0.001;** N: < **0.001;** O: < **0.001;** P: < **0.001;** Q: < **0.001;** R: < **0.001;** S: < **0.001**
F	MaxDrive 2.0 mm	408 (0.32)	3.94 (0.24)	**A: 0.026; B: < 0.001; C: < 0.001;** D: 0.987; E: 0.979; **G: < 0.001; H: < 0.001; I: < 0.001; J: < 0.001; K: < 0.001; L: < 0.001; M: < 0.001; N: < 0.001; O: < 0.001; P: < 0.001; Q: < 0.001; R: < 0.001; S: < 0.001**	408 (0.29)	22.4 (2.69)	**A: 0.002; B: < 0.001; C: < 0.001;** D: 0.570; E: > 0.999; **G: 0.001;** H: 0.051; **I: < 0.001; J: 0.001; K: 0.001; L: 0.001; M: < 0.001; N: < 0.001; O: < 0.001; P: < 0.001; Q: < 0.001; R: < 0.001; S: < 0.001**
G	Inion CPS 2.0 mm	74.5 (0.54)	0.57 (0.06)	A: 0.468; **B: < 0.001;** C: 0.436; **D: < 0.001; E: 0.001; F: < 0.001; H: 0.014;** I: 0.051; **J: 0.001; K: 0.010; L: < 0.001; M: 0.001; N: < 0.001; O: 0.001; P: 0.014; Q: < 0.001; R: < 0.001; S: < 0.001**	74.5 (0.83)	4.53 (0.35)	**A: < 0.001;** B: 0.138; **C: 0.007; D: 0.001; E: 0.001; F: 0.001; H: < 0.001;** I: 0.137; **J: 0.003;** K: 0.091; **L: 0.020; M: < 0.001; N: < 0.001; O: 0.013; P: 0.002; Q: 0.001; R: 0.003;** S: 0.060
H	Inion CPS 2.5 mm	157 (0.35)	0.82 (0.08)	A: 0.762; **B: 0.002;** C: > 0.999; **D: < 0.001; E: 0.001; F: < 0.001; G: 0.014;** I: 0.988; **J: < 0.001; K: < 0.001; L: < 0.001; M: < 0.001; N: 0.013;** O: 0.239; P: 0.999; Q: 0.252; **R: 0.013;** S: 0.062	157 (0.77)	15.8 (0.79)	**A: < 0.001; B: < 0.001; C: < 0.001; D: 0.013; E: 0.044;** F: 0.051; **G: < 0.001; I: < 0.001; J: < 0.001; K: < 0.001; L: < 0.001; M: < 0.001; N: < 0.001; O: < 0.001; P: < 0.001; Q: < 0.001; R: < 0.001; S: < 0.001**
I	LactoSorb 2.0 mm	97.6 (0.32)	0.75 (0.06)	A: 0.670; **B: < 0.001;** C: 0.996; **D: < 0.001; E: 0.001; F: < 0.001;** G: 0.051; H: 0.988; **J: < 0.001; K: < 0.001; L: < 0.001; M: < 0.001; N: 0.002; O: 0.030;** P: 0.492; **Q: 0.007; R: 0.002; S: 0.001**	97.9 (0.56)	3.76 (0.29)	**A: < 0.001;** B: > 0.999; C: 0.666; **D: 0.001; E: 0.001; F: < 0.001;** G: 0.137; **H: < 0.001; J: 0.002; K: 0.005; L: 0.003; M: < 0.001; N: < 0.001;** O: 0.568; P: 0.084; Q: 0.079; **R: 0.023;** S: 0.420
J	Macropore 2.0 mm	62.2 (0.75)	0.24 (0.02)	A: 0.218; **B: < 0.001; C: 0.033; D: < 0.001; E: 0.001; F: < 0.001; G: 0.001; H: < 0.001; I: < 0.001; K: 0.009; L: < 0.001;** M: > 0.999; **N: < 0.001; O: < 0.001; P: 0.001; Q: < 0.001; R: < 0.001; S: < 0.001**	62.2 (0.45)	8.44 (0.96)	A: > 0.999; **B: 0.001; C: 0.001; D: 0.001; E: 0.001; F: 0.001; G: 0.003; H: < 0.001; I: 0.002; K: 0.022;** L: 0.126; **M: < 0.001; N: < 0.001; O: < 0.001; P: < 0.001; Q: < 0.001; R: < 0.001; S: < 0.001**
K	Polymax 2.0 mm	58.8 (0.23)	0.37 (0.04)	A: 0.297; **B: < 0.001;** C: 0.082; **D: < 0.001; E: 0.001; F: < 0.001; G: 0.010; H: < 0.001; I: < 0.001; J: 0.009; L: < 0.001; M: 0.014; N: < 0.001; O: < 0.001; P: 0.001; Q: < 0.001; R: < 0.001; S: < 0.001**	57.5 (0.41)	5.73 (0.54)	**A: < 0.001; B: 0.002; C: 0.001; D: 0.001; E: 0.001; F: 0.001;** G: 0.091; **H: < 0.001; I: 0.005; J: 0.022;** L: 0.968; **M: < 0.001; N: < 0.001; O: < 0.001; P: < 0.001; Q: < 0.001; R: < 0.001; S: 0.003**
L	BioSorb FX 2.0 mm	81.5 (0.57)	1.55 (0.13)	A: > 0.999; **B: 0.019; C: 0.020; D: 0.001; E: 0.004; F: < 0.001; G: < 0.001; H: < 0.001; I: < 0.001; J: < 0.001; K: < 0.001; M: < 0.001; N: 0.008; O: 0.002; P: < 0.001; Q: 0.002; R: 0.011; S: 0.004**	80.9 (0.43)	6.41 (0.66)	**A: 0.003; B: 0.001; C: 0.001; D: 0.001; E: 0.001; F: 0.001; G: 0.020; H: < 0.001; I: 0.003;** J: 0.126; K: 0.968; **M: < 0.001; N: 0.001; O: < 0.001; P: < 0.001; Q: < 0.001; R: < 0.001; S: 0.001**
M	Resorb X 2.1 mm	55.9 (0.26)	0.25 (0.03)	A: 0.224; **B: < 0.001; C: 0.035; D: < 0.001; E: 0.001; F: < 0.001; G: 0.001; H: < 0.001; I: < 0.001;** J: > 0.999; **K: 0.014; L: < 0.001; N: < 0.001; O: < 0.001; P: 0.001; Q: < 0.001; R: < 0.001; S: < 0.001**	55.9 (0.30)	2.14 (0.28)	**A: < 0.001; B: 0.005; C: 0.004; D: < 0.001; E: < 0.001; F: < 0.001; G: < 0.001; H: < 0.001; I: < 0.001; J: < 0.001; K: < 0.001; L: < 0.001;** N: > 0.999; O: 0.090; P: 0.730; Q: 0.221; R: > 0.999; S: > 0.999
N	SW Rx + SP Rx 2.1 mm (D1.6/T-)	NA	1.11 (0.09)	A: 0.988; B: > 0.999; C: 0.895; **D: 0.001; E: 0.002; F: < 0.001; G: < 0.001; H: 0.013; I: 0.002; J: < 0.001; K: < 0.001; L: 0.008; M: < 0.001;** O: 0.987; P: 0.284; Q: 0.390; R: > 0.999; S: 0.745	NA	2.13 (0.28)	**A: < 0.001; B: 0.005; C: 0.004; D: < 0.001; E: < 0.001; F: < 0.001; G: < 0.001; H: < 0.001; I: < 0.001; J: < 0.001; K: < 0.001; L: < 0.001;** M: > 0.999; O: 0.086; P: 0.711; Q: 0.211; R: > 0.999; S: > 0.999
O	SW Rx + SP Rx 2.1 mm (D1.6/T2.0)	NA	1.01 (0.10)	A: 0.948; B: 0.488; C: > 0.999; **D: < 0.001; E: 0.001; F: < 0.001; G: 0.001;** H: 0.239; **I: 0.030; J: < 0.001; K: < 0.001; L: 0.002; M: < 0.001;** N: 0.987; P: 0.989; Q: > 0.999; R: 0.961; S: > 0.999	NA	3.13 (0.44)	**A: < 0.001;** B: 0.971; C: > 0.999; **D: < 0.001; E: < 0.001; F: < 0.001; G: 0.013; H: < 0.001;** I: 0.568; **J: < 0.001; K: < 0.001; L: < 0.001;** M: 0.090; N: 0.086; P: 0.998; Q: > 0.999; R: 0.208; S: 0.999
P	SW xG + SP Rx 2.1 mm (D1.6/T-)	NA	0.90 (0.11)	A: 0.862; B: 0.075; C: > 0.999; **D: < 0.001; E: 0.001; F: < 0.001; G: 0.014;** H: 0.999; I: 0.492; **J: 0.001; K: 0.001; L: < 0.001; M: 0.001;** N: 0.284; O: 0.989; Q: > 0.999; R: 0.242; S: 0.884	NA	2.71 (0.46)	**A: < 0.001;** B: 0.291; C: 0.725; **D: < 0.001; E: < 0.001; F: < 0.001; G: 0.002; H: < 0.001;** I: 0.084; **J: < 0.001; K: < 0.001; L: < 0.001;** M: 0.730; N: 0.711; O: 0.998; Q: > 0.999; R: 0.743; S: > 0.999
Q	SW xG + SP Rx 2.1 mm (D1.6/T2.0)	NA	0.97 (0.06)	A: 0.914; **B: 0.016;** C: > 0.999; **D: 0.001; E: 0.002; F: < 0.001; G: < 0.001;** H: 0.252; **I: 0.007; J: < 0.001; K: < 0.001; L: 0.002; M: < 0.001;** N: 0.390; O: > 0.999; P: > 0.999; R: 0.346; S: 0.999	NA	2.87 (0.37)	**A: < 0.001;** B: 0.398; C: 0.901; **D: < 0.001; E: < 0.001; F: < 0.001; G: 0.001; H: < 0.001;** I: 0.079; **J: < 0.001; K: < 0.001; L: < 0.001;** M: 0.221; N: 0.211; O: > 0.999; P: > 0.999; R: 0.438; S: > 0.999
R	SW xG + SP xG 2.1 mm (D1.6/T-)	NA	1.12 (0.09)	A: 0.991; B: > 0.999; C: 0.858; **D: 0.001; E: 0.002; F: < 0.001; G: < 0.001; H: 0.013; I: 0.002; J: < 0.001; K: < 0.001; L: 0.011; M: < 0.001;** N: > 0.999; O: 0.961; P: 0.242; Q: 0.346; S: 0.666	NA	1.86 (0.67)	**A: < 0.001; B: 0.036;** C: 0.097; **D: < 0.001; E: < 0.001; F: < 0.001** **G: 0.003; H: < 0.001; I: 0.023; J: < 0.001; K: < 0.001; L: < 0.001;** M: > 0.999; N: > 0.999; O: 0.208; P: 0.743; Q: 0.438; S: 0.995
S	SW xG + SP xG 2.1 mm (D1.6/T2.0)	NA	1.01 (0.04)	A: 0.943; **B: 0.037;** C: > 0.999; **D: 0.001; E: 0.002; F: < 0.001; G: < 0.001;** H: 0.062; **I: 0.001; J: < 0.001; K: < 0.001; L: 0.004; M: < 0.001;** N: 0.745; O: > 0.999; P: 0.884; Q: 0.999; R: 0.666	NA	2.58 (0.82)	**A: < 0.001;** B: 0.654; C: 0.933; **D: < 0.001; E: < 0.001; F: < 0.001;** G: 0.060; **H: < 0.001;** I: 0.420; **J: < 0.001; K: 0.003; L: 0.001;** M: > 0.999; N: > 0.999; O: 0.999; P: > 0.999; Q: > 0.999; R: 0.995

**Figure 5:**
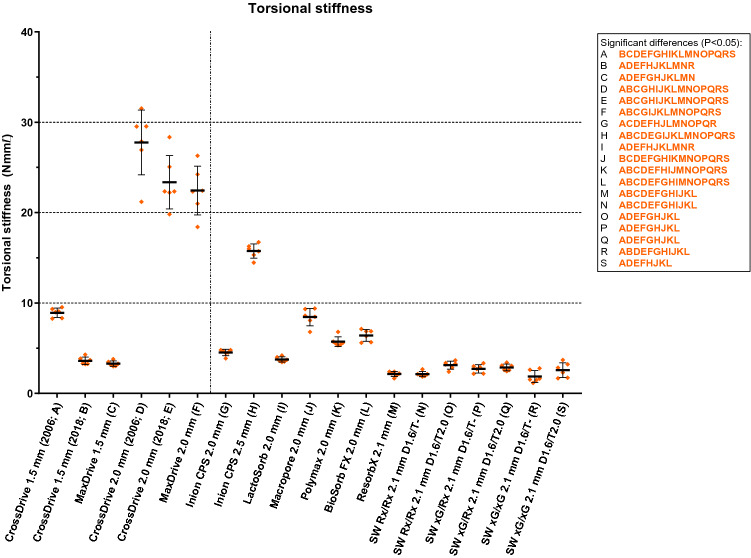
The torsional stiffness of all included osteosynthesis systems. The characters in orange represent significant differences in stiffness (Nmm/°). *D, *drill diameter (mm); *T, *tap diameter (mm). *Error bars: *mean values ± standard deviation. The dotted line separates the titanium (left) and biodegradable systems (right). All the stiffness values, including the P-values of the pairwise comparisons, are reported in Table 3.

*Ref* reference, also used in the pairwise comparison column and in Figs. 4 and 5; *SD* standard deviation, *NA* not applicable. The bold P-values represent the statistically significant values after correcting for multiple testing (P < 0.05).

The mean torsional stiffness of the titanium 2.0 mm systems was significantly higher compared to the 1.5 mm titanium systems (Fig. 5 and Table 3). Of all the biodegradable systems, the Inion CPS 2.5 mm had the highest torsional stiffness (15.8 (0.79) Nmm/°). There were no significant differences in torsional stiffness between the SonicWeld Rx and xG systems. The torsional stiffness of the SonicWeld systems was similar to the Resorb X system. None of the osteosynthesis systems fractured during the torsion tests.

## Discussion

The aim of this study was to be able to guide surgeons in the selection of osteosynthesis systems. We determined and compared the mechanical properties of biodegradable and titanium osteosynthesis systems used in OMF-surgery. The pull-out load of the SonicPins Rx and xG systems was comparable, irrespective of whether the burr hole was tapped or not. The CrossDrive (2018) and MaxDrive titanium systems demonstrated lower tensile and torsional stiffness accompanied with higher ductility than the corresponding CrossDrive (2006) systems. The side bending stiffness of the 1.5 mm titanium systems was comparable to, and that of the 2.0 mm systems was higher than, the biodegradable systems. Regarding the biodegradable systems, the Inion CPS 2.5 mm had the highest tensile load and torsional stiffness, all the SonicWeld 2.1 mm systems had the highest tensile stiffness, and the BioSorbFX 2.0 mm had highest side bending stiffness. On the basis of the results of this study recommendations are made and discussed below which biodegradable and titanium osteosynthesis systems are preferably used for fracture and osteotomy fixation in OMF-surgery (Table 4).

**Table 4:** Proposal for recommended titanium and biodegradable osteosynthesis systems for specific indications.

Indications	Titanium systems	Biodegradable systems
Midface fractures (e.g., zygomatic or maxillary fractures) and osteotomies (e.g., Le Fort I osteotomy)	MaxDrive or CrossDrive (2018) 1.5 mm^a^	SonicWeld Rx/Rx 2.1 mm or BioSorb FX 2.0 mm
Fractures where high torsional forces are expected (e.g., mandibular symphysis fractures)	MaxDrive or CrossDrive (2018) 2.0 mm^a^	Inion CPS 2.5 mm^b^ BioSorb 2.0 mm^b^
Mandibular osteotomies (e.g. bilateral sagittal split osteotomy) and non-load bearing mandibular fractures other than symphysis fractures	MaxDrive or CrossDrive (2018) 2.0 mm^a^	Inion CPS 2.5 mm^c^

Note that the recommendations are made based on the tested osteosynthesis systems.

^a^There is no clinically relevant mechanical difference between the CrossDrive (2018) and MaxDrive systems.

^b^The Inion CPS 2.5 mm system has the most favourable mechanical properties, but whenever the bulkiness of this system is considered an issue, the BioSorb FX 2.0 mm is a suitable alternative (i.e., − 58% in volume).

^c^This is the only biodegradable system that is certified for the specific indication and that could be tested in this study (i.e., OsteotransMX mechanical properties were insufficient to be tested in this study).

The mechanical properties of osteosynthesis systems depend on several factors including composition (i.e., titanium (alloys) or (co-)polymers), the production processes of titanium systems (e.g., stamping versus laser cutting)^31–33^, dimensions, self-reinforcing of polymers^34^, the application method (i.e., screws or thermoplastic pins)^12^, ageing, and sterilization methods^35–37^. Self-reinforcing polymers is a manufacturing technique whereby the polymers are orientated in reinforcing units, such as fibrils or fibers, and the binding matrix has the same chemical structure^34^. This high degree of molecular orientation results in improved mechanical properties compared to identical polymers and dimensions^38^.

The pull-out loads of SonicPins Rx and xG were comparable, but tapping the SonicPins Rx burr hole lowered the pull-out stiffness. This could be due to the fact that tapping the burr hole increases the volume of the burr hole, while the pin’s volume remains the same. Therefore, the density of the pin in the burr hole is lowered which then decreases the stiffness. This indicates that the volume of the SonicPin Rx in the burr hole is a limiting factor that lowers the pull-out stiffness compared to not tapping the burr hole. On the other hand, this effect did not occur with the SonicPin xG indicating that, although the volume of the SonicPin xG in the burr hole is also lowered, compared to not tapping the burr hole, the stiffness of the copolymer itself is sufficient to sustain the pull-out stiffness.

The plate and screw dimensions are important characteristics when evaluating mechanical properties of osteosynthesis systems. In particular, the tensile load and torsional stiffness increase significantly when the cross sectional area increases as shown by the results of the Inion CPS 2.0 versus 2.5 mm systems^39^. The effects of self-reinforcing polymers is demonstrated by the differences in the mechanical properties of the BioSorb FX 2.0 mm (SR 70/30 PLLA/PDLLA) and Polymax 2.0 mm (70/30 PLLA/PDLLA) systems. Furthermore, the effect of the application method (i.e., melting of thermoplastic pins in the plates compared to usage of screws) is demonstrated by the mechanical properties of the Resorb X (100% PDLLA with screws) compared to the SonicWeld Rx/Rx (100% PDLLA with thermoplastic pins) systems. Additionally, the origin of the failure of all the SonicWeld systems shows that melting the pins within the plates causes a shift of the weakest link of the complete osteosynthesis system from the screw-plate interface (i.e., all other biodegradable systems) to the plate itself (i.e., SonicWeld and titanium systems).

Several studies have assessed the mechanical forces surrounding osteosyntheses applied to maxillofacial fractures^28,40–44^, osteotomies^45,46^ and reconstructions^47^. After maxilofacial trauma, the reported bite force at fracture fixation increases up to 64 N by the second postoperative day, 92 N after 1 week, 187 N after 4 weeks, and to 373 N at the 3-month follow-up^40^. Other studies focusing on trauma patients showed that 100 N forces were measured after 4 weeks of fixation^41,43^. The mechanical forces around maxillofacial osteotomies have been reported to increase from 21 ± 14 N (i.e., after 1 week) to 65 ± 43 N (i.e., after 6 weeks)^42^ while other studies report forces ranging from 82.5 to 132N^45,46^. The masticatory forces after mandibular reconstructions range from 28 to 186N^47^. These reported data indicate that the mechanical properties of all the titanium and most of the biodegradable osteosynthesis systems are sufficient for adequate fixation. However, the mechanical stress surrounding osteosynthesis systems is multi-factorial and is affected by the location of the fracture, differences in interfragmentary stability (i.e., of fractures), mandibulair height (i.e., following fractures, osteotomies and reconstructions), degree and direction of movement (i.e., after an osteotomy), and preoperative mastericatory forces^29,41,48^. Additionally, as bone healing progresses, the forces will be shared by the osteosynthesis system and the underlying bone. Therefore, it remains difficult to estimate the least mechanical properties an osteosynthesis system has to meet.

Although high mechanical osteosynthesis properties are sought for adequate fixation, the extreme stiffness of the titanium systems is a disadvantage due to the stress shielding of the underlying bone^4^. A remarkable reduction in tensile, side bending, and torsional stiffnesses of the CrossDrive (2018) and MaxDrive compared to the CrossDrive (2006) systems was observed, while their tensile loads were comparable. The reduction in stiffnesses is the result of an adapted production process of the newer CrossDrive (2018) and MaxDrive systems compared to the older CrossDrive (2006) system. In 2007, the production process of the KLS Martin titanium systems was altered from stamping (also known as metal pressing) to milling of plates. Differences in manufacturing processes (e.g., heat treatment during stamping of plates) are known to alter the mechanical properties of titanium^16,31,32,49–51^. The reduction in stiffness may be beneficial as this may reduce bone stress shielding and thus should be assessed in vivo in future research. Additionally, the CrossDrive (2018) and MaxDrive showed higher ductility compared to the CrossDrive (2006) systems. This is also preferred since it demonstrates that the CrossDrive (2018) and MaxDrive plates undergo more plastic deformation compared to the CrossDrive (2006) plates before fracturing. The CrossDrive (2018) and MaxDrive systems still meet the ASTM and ISO standard requirements for surgical titanium implants^52–55^. Additionally, this study shows that the newer CrossDrive (2018) and MaxDrive osteosynthesis systems remain to have higher mechanical properties than the tested biodegradable osteosynthesis systems. However, as clinical studies have shown that biodegradable and titanium osteosynthesis systems have similar efficacy in maxillofacial traumatology^9^, both systems have mechanical properties that suffice for clinical application.

Three important aspects have to be noted before recommendations for clinical use can be made. First, it must be noted that statistical differences do not imply clinically relevant differences. Second, the stiffness of an osteosynthesis system is a more clinically relevant outcome than load since this affects adequate fixation and bone healing (i.e., malunion and non-union)^56^ while tensile load is only relevant whenever the bone segments have been separated by more than a few millimeters. In the latter case, this will certainly result in compromised bone healing or malunion. Thirdly, although extreme tensile stiffness is a concern in titanium systems due to stress shielding, it is not a concern when using biodegradable systems as they undergo bulk degradation thereby decreasing their mechanical properties with time^13^.

Our study aimed to guide OMF-surgeons in the selection of titanium and biodegradable osteosynthesis systems. The CrossDrive (2018) and MaxDrive 1.5 mm titanium systems are recommended for midface fractures (e.g., zygomatic or maxillary fractures) and osteotomies (e.g., Le Fort I osteotomy), and the CrossDrive (2018) and MaxDrive 2.0 mm titanium systems for mandibular fractures and osteotomies when a titanium osteosynthesis system is used (Table 4). The CrossDrive (2018) or MaxDrive systems are prescribed over the CrossDrive (2006) system as all tested titanium systems meet the ASTM and ISO standard requirements for surgical titanium implants while the higher ductality and lower stiffness of the CrossDrive (2018) and MaxDrive could be benificial in clinical use. The reduction in stiffness may reduce stress shielding of the underlying bone, although further in vivo research is necessary to prove this. There is no clinically relevant mechanical difference between the CrossDrive (2018) and MaxDrive systems. The manufacturer states that the adapted screw head of the MaxDrive system could result in better perioperative handling by surgeons, but this still has to be assessed objectively.

When there is an indication for a biodegradable osteosynthesis system, the SonicWeld Rx/Rx 2.1 mm or BioSorbFX 2.0 mm systems are recommended to fixate midface fractures (e.g., zygomatic or maxillary fractures) and osteotomies (e.g., Le Fort I osteotomies) due to their high tensile and side bending stiffness, respectively (Table 4)^57,58^. Both systems have their own advantages regarding perioperative handeling, viz., the possibility to adapt the BioSorb FX plate at room temperature^34^ and the avoidance of tapping the burr holes when using the SonicWeld system^57^. When also considering the dimensions and volumes, and the (co-)polymer compositions of these two systems, the SonicWeld Rx/Rx system is preferred as it is less bulky (i.e., − 14% in volume) and has a more favourable degradative copolymer composition^13,59^. Whenever low pull-out forces are expected, we recommend not tapping the SonicPins burr holes as this remains an extra perioperative, time-consuming step for surgeons^9^. On the other hand, whenever high torsional forces are expected (e.g., fixating mandibular symphysis fractures^41^), the Inion CPS 2.5 mm system is recommended although it might be bulky due to the plate and screw dimensions. Whenever the bulkiness of this system is considered an issue (e.g., complicating stress free closure of the incision or due to palpability), the BioSorb FX 2.0 mm is a suitable alternative (i.e., − 58% in volume; Table 4). Only two of the tested biodegradable osteosynthesis systems (i.e., Inion CPS 2.5 mm and OsteotransMX 2.0 mm with plate thickness of 1.4 mm) are certified to be used for mandibular osteotomies and non-load bearing mandibular fractures other than mandibular symphysis fractures^59,60^. Therefore, as the OsteotransMX 2.0 mm has insufficient mechanical properties to be tested in our study, we recommend the Inion CPS 2.5 mm system for fixation of mandibular osteotomies (e.g., bilateral sagittal split osteotomy) and non-load bearing mandibular fractures other than symphysis fractures (Table 4). However, it must be noted that, although the mechanical propterties of the Inion CPS 2.5 mm system are sufficient for for fixation of mandibular osteotomies, a randomized controlled trial reported a significantly higher symptomatic plate removal risk of the Inion CPS 2.5 mm compared to the CrossDrive (2018) 2.0 mm titanium system after fixation of BSSOs^2^. Therefore, when choosing an appropriate osteosynthesis system for fixation of mandibular osteotomies, we recommend OMF-surgeons to take both aspects (i.e., the mechanical properties and the risk of symptomatic plate removal) into account.

This is the most comprehensive study to date comparing various commonly used titanium and biodegradable osteosynthesis systems in OMF-surgery. Other strengths of this study are the standardized and reproducable osteosynthesis systems application methods (e.g., screws inserted using the mean applied torque by four experienced OMF-surgeons), usage of standardized test setups, and the assessment of a variety of outcomes that are relevant to clinical practice (i.e., tensile and pull-out load, and tensile, pull-out, side-bending, and torsional stiffness). Additionally, to ensure comparability, all the osteosynthesis systems were applied by one researcher (BG) while the tests were performed by another researcher (CCR). Furthermore, PMMA was chosen instead of bone blocks for all the tests because the variability in bone mineral density, in cortical and spongious bone layer thickness, and in block dimensions impede the latter’s use as a standardized and reproducible model as these factors may confound the results of the mechanical tests. The mechanical properties of PMMA are similar to bone^22–24^, the quality of each PMMA-block is similar (i.e., no variability in density), and blocks with identical dimensions can be easily fabricated, which ensures standardization and reproducibility of the test setups.

A limitation of this study is that the insertion of the SonicPins could not be quantified, as was done with the torque applied to the screws, because the pins are melted into the burr holes. We tried to address this by having one researcher (BG) insert the pins and by using a minimum and maximum amount of time (i.e., one and two seconds per pin, respectively) as a quantifying measure of insertion. Furthermore, the Osteotrans-MX system could not be tested due to the screw heads failing before the screws were fully screwed in. This indicates that PMMA is not suitable for testing the screws’ mechanical properties. Using allo- or xenograft bone may address this limitation. However, we did not perform tests in bone due to the abovementioned limitations of bone and the fact that all the other osteosynthesis systems could be tested in PMMA, thus ensuring standardization and reproducibility of the test setups.

In conclusion, this study shows that the pull-out load and stiffness of SonicPinx Rx and xG are comparable and that tapping the burr hole does not improve the pull-out load and stiffness significantly. Furthermore, the KLS CrossDrive (2018) and MaxDrive titanium systems have significantly lower tensile and torsional stiffness, combined with higher ductility, than the corresponding CrossDrive (2006) titanium systems, while maintaining similar tensile load. The reduction in stiffness may reduce stress shielding of the underlying bone, although the clinical relevance of the reduction in stiffness was not investigated in this study. On the basis of the results of this study, the CrossDrive (2018) and MaxDrive 1.5 mm titanium systems are recommended for midface fractures (e.g., zygomatic or maxillary fractures) and osteotomies (e.g., Le Fort I osteotomy), and the CrossDrive (2018) and MaxDrive 2.0 mm titanium systems for mandibular fractures and osteotomies when a titanium osteosynthesis system is used. When there is an indication for a biodegradable osteosynthesis system, the SonicWeld 2.1 mm or BioSorbFX 2.0 mm are recommended for midface fractures and osteotomies, and the Inion CPS 2.5 mm biodegradable system for mandibular osteotomies and non-load bearing mandibular fractures, especially when high torsional forces are expected (e.g., mandibular symphysis fractures).

## Supplementary information

Supplementary Information.

## Data Availability

The materials and datasets used and analysed during the present study are available from the corresponding author on reasonable request.
