# Supplementary material for: Comparison of the mechanical properties of biodegradable and titanium osteosynthesis systems used in oral and maxillofacial surgery
Source: Sci Rep. 2020 Oct 23;10:18143. doi: 10.1038/s41598-020-75299-9 (PMC7584639; doi:10.1038/s41598-020-75299-9)
Supplement: Supplementary file 1 — Supplementary Information. [file 41598_2020_75299_MOESM1_ESM.pdf]

Supplementary files

Comparison of the mechanical properties of biodegradable and titanium osteosynthesis systems used in oral and maxillofacial surgery

Barzi Gareb*[1], Charlotte C. Roossien[2], Nico B. van Bakelen[1], Gijsbertus J. Verkerke[3], Arjan Vissink[1], Ruud R.M. Bos[1], Baucke van Minnen[1]

Institutions

[1]University of Groningen, University Medical Center Groningen, Department of Oral and Maxillofacial Surgery, Hanzeplein 1, 9713 GZ Groningen, The Netherlands, P.O. Box 30001, 9700 RB Groningen, The Netherlands.

[2]University of Groningen, University Medical Center Groningen, Department of Rehabilitation Medicine, Hanzeplein 1, 9713 GZ, Groningen, The Netherlands, P.O. Box 30001, 9700 RB Groningen, The Netherlands.

[3]University of Groningen, University Medical Center Groningen, Department of Rehabilitation Medicine, Hanzeplein 1, 9713 GZ, Groningen, The Netherlands, P.O. Box 30001, 9700 RB Groningen, The Netherlands; University of Twente, Department of Biomechanical Engineering, Drienerlolaan 5, 7522 NB, Enschede, the Netherlands.

Corresponding author

Barzi Gareb
P.O. Box 30001, 9700 RB Groningen, The Netherlands
Telephone number: +31 503611054
Fax number: +31 503612831
E-mail address: b.gareb@umcg.nl

Supplementary Tables

Supplementary Table S1: The pull-out load and stiffness of SonicPins Rx and xG without and with tapping the burr holes.

Supplementary Table S2: The torque applied until hand-tight fixation (i.e., the mean of four experienced oral and maxillofacial surgeons) and until screw breakage of all the included osteosynthesis systems.

Supplementary Figures

Supplementary Figure S1: The torque applied until hand-tight fixation (i.e., the mean of four experienced oral and maxillofacial surgeons) and until screw breakage of all the included osteosynthesis systems. The characters in green and orange represent significant differences in maximum torque (Nmm) until hand-tight fixation and until breakage, respectively. *Error bars: mean values ± standard deviation. The dotted line separates the titanium (left) and biodegradable systems (right). All the values, including the P-values of the pairwise comparisons, are reported in Supplementary Table S2.*

Supplementary Figure S2: Force-displacement graphs derived from the tensile test of the KLS SonicWeld Rx osteosynthesis system. The blue (n=6) and red lines (n=6) represent the results from our previous[1] and current studies, respectively.

Supplementary Figure S3: Force-displacement graphs derived from the side bending test of the KLS SonicWeld Rx osteosynthesis system. The blue (n=6) and red lines (n=6) represent the results from our previous[1] and current studies, respectively.

Supplementary Figure S4: Force-displacement graphs derived from the torsion test of the KLS SonicWeld Rx 2.1mm osteosynthesis system. The blue (n=6) and red lines (n=6) represent the results from our previous[1] and current studies, respectively.

Supplementary Figure S5: Force-displacement graphs derived from the tensile test of the KLS MaxDrive, CrossDrive (2006), and CrossDrive (2018) 1.5 mm osteosynthesis systems, indicating higher ductility of the CrossDrive (2018) and MaxDrive systems compared to the CrossDrive (2006) system.

Supplementary Figure S6: Force-displacement graphs derived from the tensile test of the KLS MaxDrive, CrossDrive (2006), and CrossDrive (2018) 2.0 mm osteosynthesis systems, indicating higher ductility of the CrossDrive (2018) and MaxDrive systems compared to the CrossDrive (2006) system.

Supplementary Table S1: The pull-out load and stiffness of SonicPins Rx and xG without and with tapping the burr holes.

				Maximum load		Stiffness	
**Ref**	**System**	**Drill (mm)**	**Tap (mm)**	**Mean Fmax (SD) in N**	**P-values (pairwise comparison)**	**Mean stiffness (SD) in N/mm**	**P-values (pairwise comparison)**
1	SonicPin Rx 2.1mm	1.6	None	55.5 (14.5)	**2: 0.001;** 3: 0.539; 4: 0.474; 5: >0.999; 6: 0.423	117 (7.14)	**2: 0.002;** 3: 0.278; **4: 0.024;** 5: >0.999; 6: >0.999
2	SonicPin Rx 2.1mm	1.6	1.7	29.7 (7.08)	**1: 0.001;** 3: 0.304; **4: <0.001; 5: <0.001; 6: <0.001**	93.4 (6.53)	**1: 0.002;** 3: 0.774; 4: 0.468; **5: 0.020; 6: 0.001**
3	SonicPin Rx 2.1mm	1.6	1.8	43.3 (11.1)	1: 0.539; 2: 0.304; **4: 0.002;** 5: 0.316; **6: 0.001**	103 (12.1)	1: 0.278; 2: 0.774; 4: >0.999; 5: 0.390; 6: 0.260
4	SonicPin Rx 2.1mm	1.6	2.0	68.0 (6.94)	1: 0.474; **2: 0.001; 3: 0.002;** 5: 0.793; 6: >0.999	101 (6.25)	**1: 0.024;** 2: 0.468; 3: >0.999; 5: 0.137; **6: 0.010**
5	SonicPin xG 2.1mm	1.6	None	56.8 (9.50)	1: >0.999; **2: <0.001;** 3: 0.316; 4: 0.793; 6: 0.712	118 (11.4)	1: >0.999; **2: 0.020;** 3: 0.390; 4: 0.137; 6: >0.999
6	SonicPin xG 2.1mm	1.6	2.0	68.3 (5.83)	1: 0.423; **2: <0.001; 3: 0.001;** 4: >0.999; 5: 0.712	117 (5.05)	1: >0.999; **2: 0.001;** 3: 0.260; **4: 0.010;** 5: >0.999

Ref, reference, also used in the column for pairwise comparisons and in Fig. 2; SD, standard deviation. The bold P-values represent the statistically significant values after correcting for multiple testing ($P<0.05$).

Supplementary Table S2: The torque applied until hand-tight fixation (i.e., the mean of four experienced oral and maxillofacial surgeons) and until screw breakage of all the included osteosynthesis systems.

		Hand tight		Break	
**Ref.**	**System**	**Mean torque (SD) in Nmm**	**P-values (pairwise comparison)**	**Mean torque (SD) in Nmm**	**P-values (pairwise comparison)**
A	CrossDrive 1.5 mm	247 (89.1)	**B: 0.046; C: 0.002; D: 0.001; E: <0.001; F: <0.001; G: <0.001; H: <0.001; I: <0.001; J: <0.001; K: <0.001**	396 (9.00)	**B: <0.001; C: <0.001; D: <0.001; E: <0.001; F: <0.001; G: <0.001; H: <0.001; I: <0.001; J: <0.001; K: <0.001**
B	MaxDrive 1.5 mm	319 (65.2)	**A: 0.046;** C: 0.951; D: 0.306; **E: <0.001; F: <0.001; G: <0.001; H: <0.001; I: <0.001; J: <0.001; K: <0.001**	528 (16.9)	**A: <0.001; C: <0.001; D: <0.001; E: <0.001; F: <0.001; G: <0.001; H: <0.001; I: <0.001; J: <0.001; K: <0.001**
C	CrossDrive 2.0 mm	367 (122)	**A: 0.002;** B: 0.951; D: >0.999; **E: <0.001; F: <0.001; G: <0.001; H: <0.001; I: <0.001; J: <0.001; K: <0.001**	>680	A: **<0.001;** B: **<0.001;** D: **-;** E: **<0.001;** F: **<0.001;** G: **<0.001;** H: **<0.001;** I: **<0.001;** J: **<0.001;** K: **<0.001**
D	MaxDrive 2.0 mm	407 (138)	**A: 0.001;** B: 0.306; C: >0.999; **E: <0.001; F: <0.001; G: <0.001; H: <0.001; I: <0.001; J: <0.001; K: <0.001**	>680	A: **<0.001;** B: **<0.001;** C:-; E: **<0.001;** F: **<0.001;** G: **<0.001;** H: **<0.001;** I: **<0.001;** J: **<0.001;** K: **<0.001**
E	Inion CPS 2.0 mm	73.4 (12.2)	**A: <0.001; B: <0.001; C: <0.001; D: <0.001; F: <0.001; G: <0.001; H: 0.005; I: <0.001;** J: 0.999; **K: <0.001**	85.1 (12.3)	**A: <0.001; B: <0.001; C: <0.001; D: <0.001; F: <0.001; G: <0.001;** H: 0.950; I: >0.999; **J: <0.001;** K: 0.839
F	Inion CPS 2.5 mm	157 (18.0)	**A: <0.001; B: <0.001; C: <0.001; D: <0.001; E: <0.001; G: <0.001; H: <0.001; I: <0.001; J: <0.001; K: <0.001**	181 (5.49)	**A: <0.001; B: <0.001; C: <0.001; D: <0.001; E: <0.001;** G: 0.998; **H: <0.001; I: <0.001;** J: 0.826; **K: <0.001**
G	LactoSorb 2.0 mm	96.9 (23.5)	**A: <0.001; B: <0.001; C: <0.001; D: <0.001; E: <0.001; F: <0.001; H: <0.001; I: <0.001;** J: 0.261; **K: <0.001**	189 (15.7)	**A: <0.001; B: <0.001; C: <0.001; D: <0.001; E: <0.001;** F: 0.998; **H: <0.001; I: <0.001;** J: >0.999; **K: <0.001**
H	Macropore 2.0 mm	61.7 (10.2)	**A: <0.001; B: <0.001; C: <0.001; D: <0.001; E: 0.005; F: <0.001; G: <0.001;** I: 0.994; **J: 0.009;** K: 0.688	77.2 (5.05)	**A: <0.001; B: <0.001; C: <0.001; D: <0.001;** E: 0.950; **F: <0.001; G: <0.001;** I: 0.175; **J: <0.001;** K: >0.999
I	Polymax 2.0 mm	56.7 (14.3)	**A: <0.001; B: <0.001; C: <0.001; D:**	89.5 (8.92)	**A: <0.001; B: <0.001; C: <0.001; D:**

			**<0.001; E: <0.001; F: <0.001; G: <0.001;** H: 0.994; **J: 0.001;** K: >0.999		**<0.001;** E: >0.999; **F: <0.001; G: <0.001;** H: 0.175; **J: <0.001;** K: 0.223
J	BioSorb FX 2.0 mm	80.2 (23.4)	**A: <0.001; B: <0.001; C: <0.001; D: <0.001;** E: 0.999; **F: <0.001;** G: 0.261; **H: 0.009; I: 0.001; K: <0.001**	192 (14.2)	**A: <0.001; B: <0.001; C: <0.001; D: <0.001; E: <0.001;** F: 0.826; G: >0.999; **H: <0.001; I: <0.001; K: <0.001**
K	ResorbX 2.1 mm	55.4 (11.5)	**A: <0.001; B: <0.001; C: <0.001; D: <0.001; E: <0.001; F: <0.001; G: <0.001;** H: 0.688; I: >0.999; **J: <0.001**	82.9 (11.9)	**A: <0.001; B: <0.001; C: <0.001; D: <0.001;** E: 0.839; **F: <0.001; G: <0.001;** H: >0.999; I: 0.223; **J: <0.001**

Ref, reference, also used in the pairwise comparisons column and in Supplementary Fig. S1; SD, standard deviation.
The bold P-values represent statistically significant values after correcting for multiple testing (P<0.05).

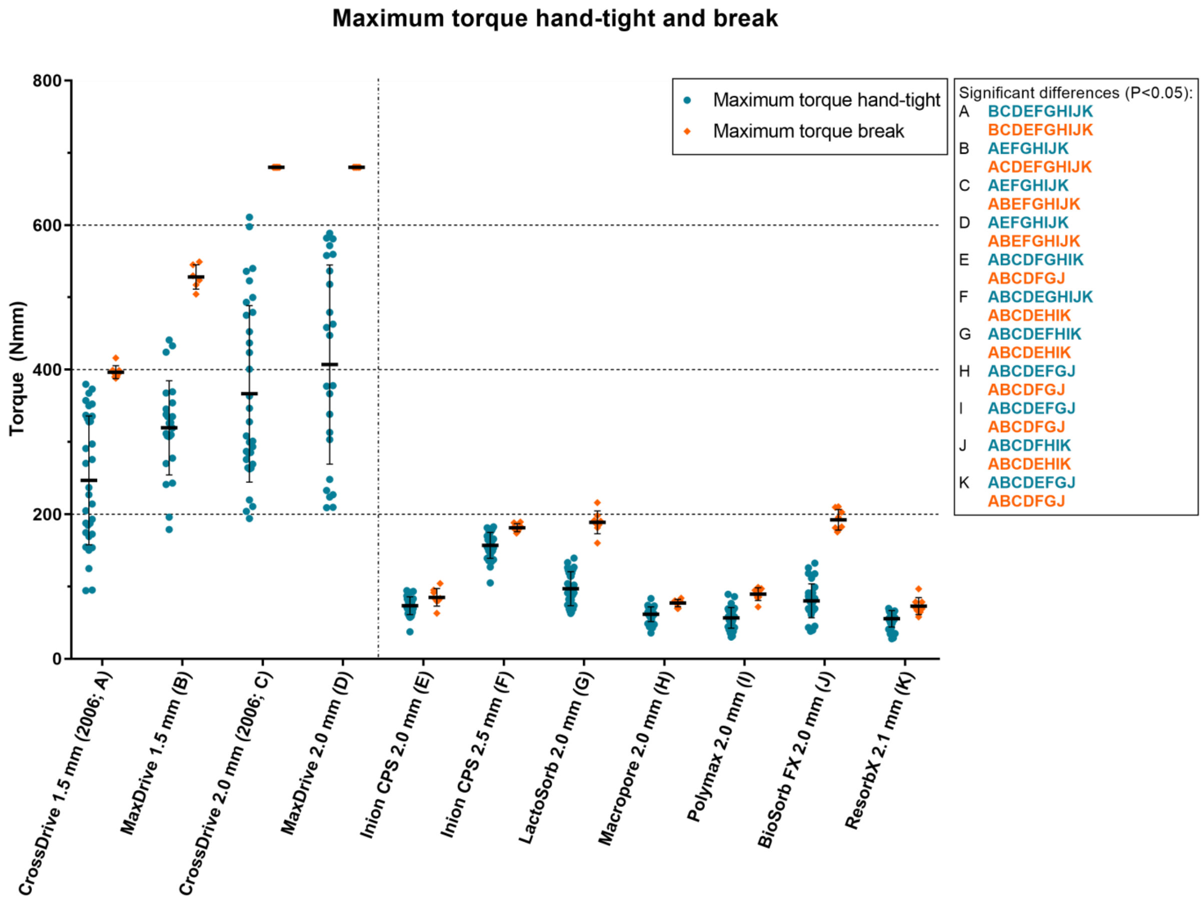

Supplementary Figure S1: The torque applied until hand-tight fixation (i.e., the mean of four experienced oral and maxillofacial surgeons) and until screw breakage of all the included osteosynthesis systems. The characters in green and orange represent the significant differences in maximum torque (Nmm) until hand-tight fixation and until breakage, respectively. *Error bars: mean values ± standard deviation. The dotted line separates the titanium (left) and biodegradable systems (right). All the values, including the P-values of the pairwise comparisons, are reported in Supplementary Table S2.*

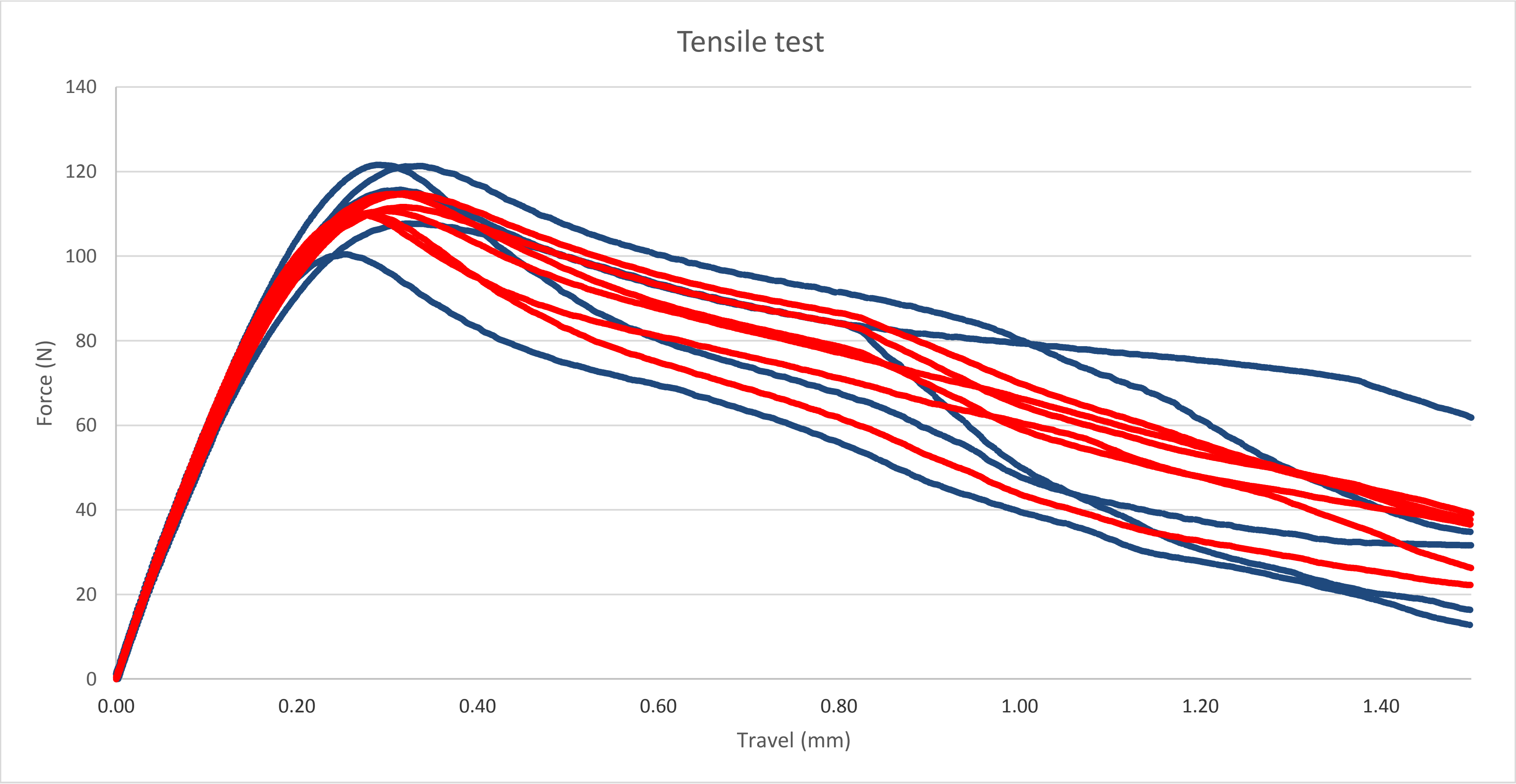

Supplementary Figure S2: Force-displacement graphs derived from the tensile test of the KLS SonicWeld Rx osteosynthesis system. The blue (n=6) and red lines (n=6) represent the results from our previous[1] and current studies, respectively.

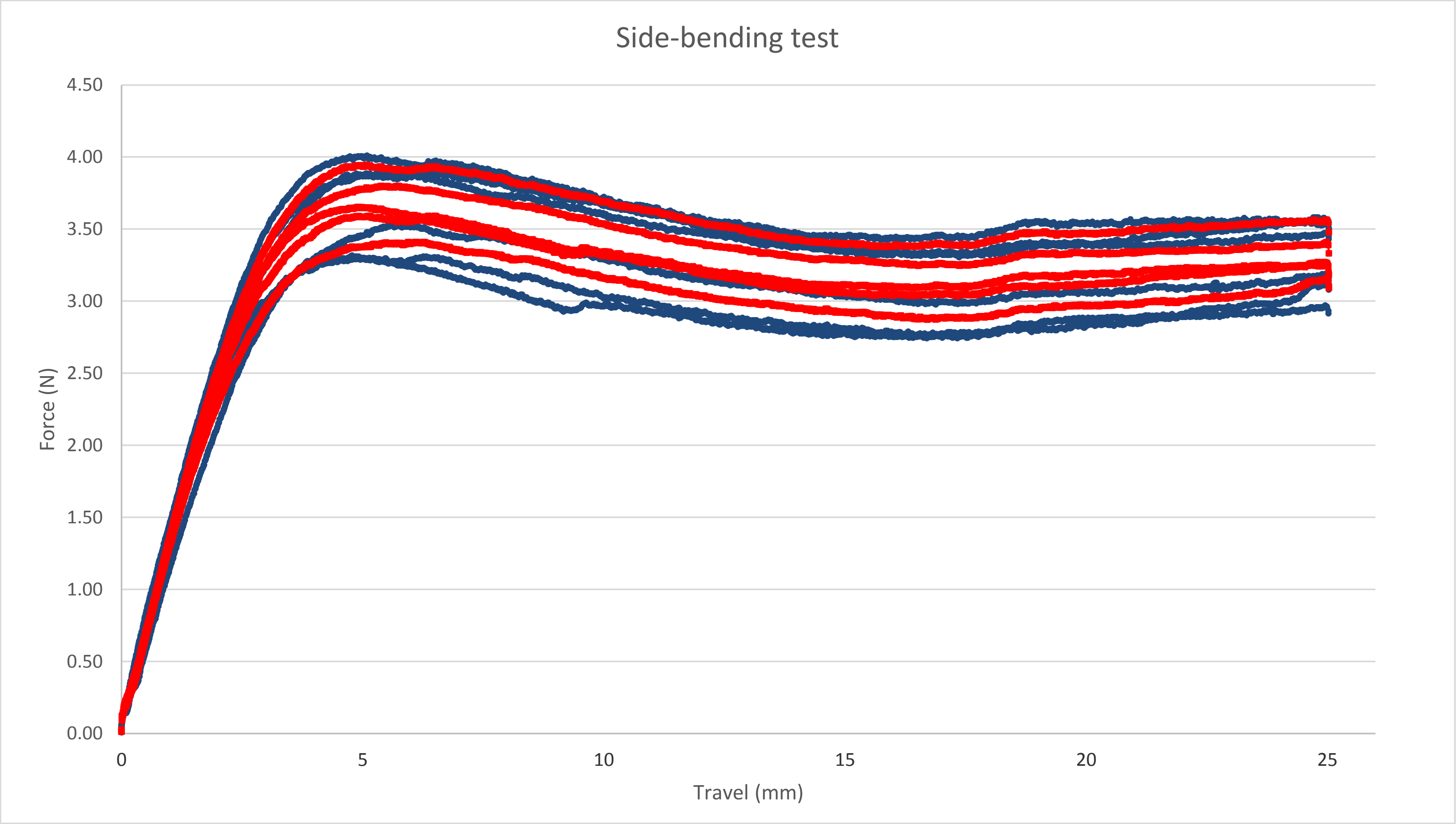

Supplementary Figure S3: Force-displacement graphs derived from the side bending test of the KLS SonicWeld Rx osteosynthesis system. The blue (n=6) and red lines (n=6) represent the results from our previous[1] and current studies, respectively.

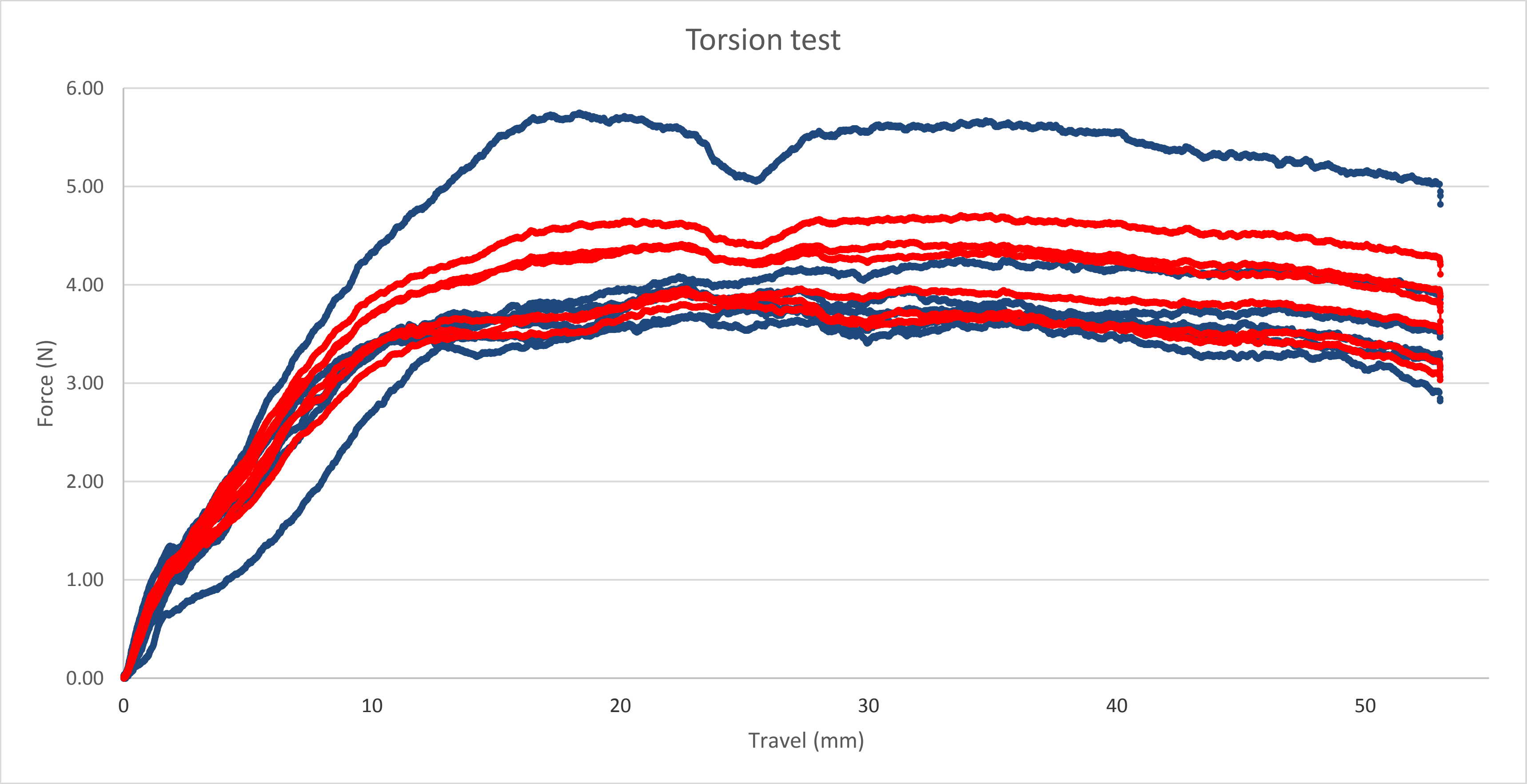

Supplementary Figure S4: Force-displacement graphs derived from the torsion test of the KLS SonicWeld Rx 2.1mm osteosynthesis system. The blue (n=6) and red lines (n=6) represent the results from our previous[1] and current studies, respectively.

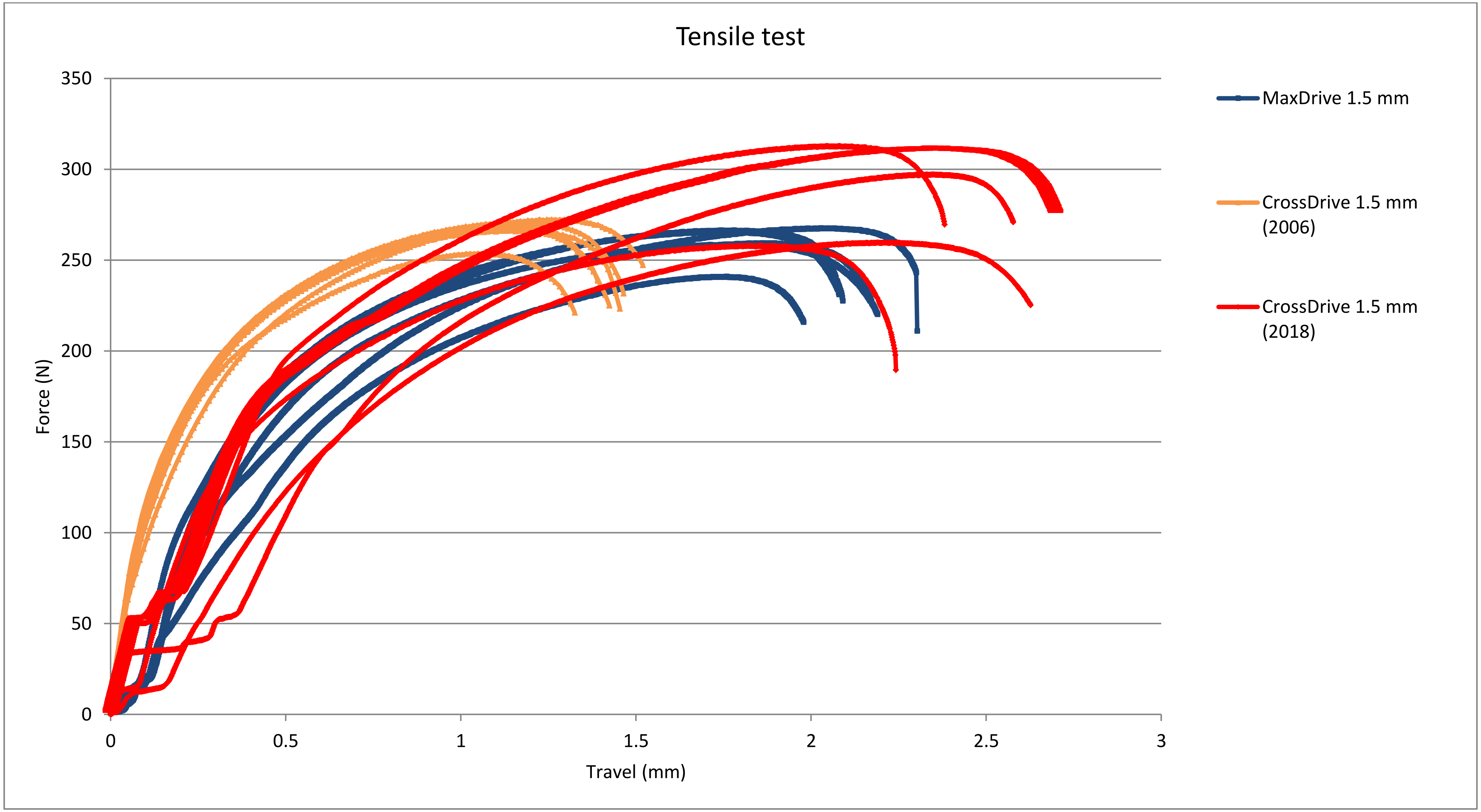

Supplementary Figure S5: Force-displacement graphs derived from the tensile test of the KLS MaxDrive, CrossDrive (2006), and CrossDrive (2018) 1.5 mm osteosynthesis systems, indicating higher ductility of the CrossDrive (2018) and MaxDrive systems compared to the CrossDrive (2006) system.

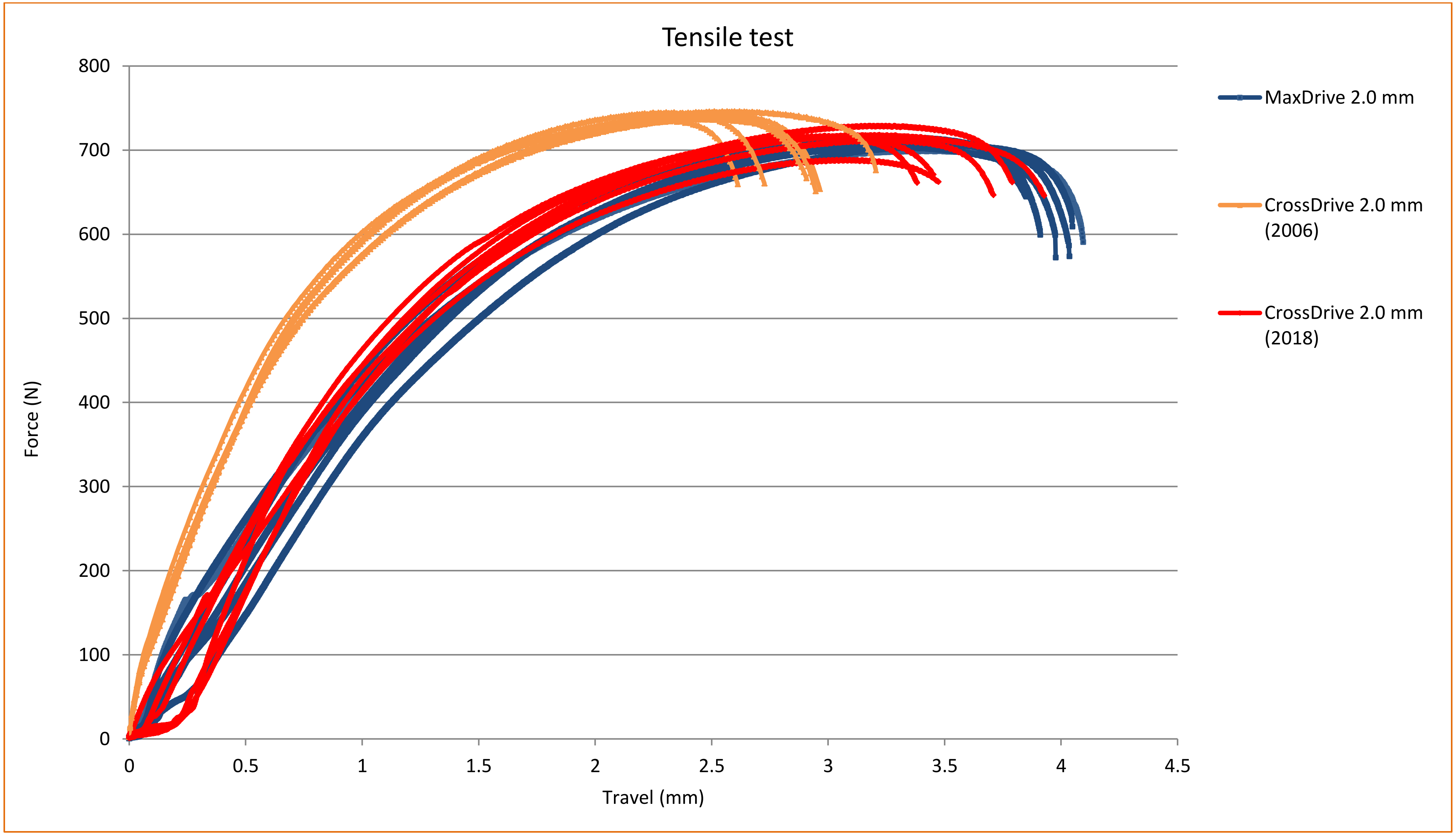

Supplementary Figure S6: Force-displacement graphs derived from the tensile test of the KLS MaxDrive, CrossDrive (2006), and CrossDrive (2018) 2.0 mm osteosynthesis systems, indicating higher ductility of the CrossDrive (2018) and MaxDrive systems compared to the CrossDrive (2006) system.

Reference

1. Buijs, G. J., van der Houwen, E. B., Stegenga, B., Verkerke, G. J. & Bos, R. R. M. Mechanical Strength and Stiffness of the Biodegradable SonicWeld Rx Osteofixation System. *J. Oral Maxillofac. Surg.* **67**, 782–787 (2009).
